# Disentangling non-specific and condition-specific transcriptomic responses: A framework from heat stress in yeast

**DOI:** 10.1016/j.crmicr.2026.100649

**Published:** 2026-07-20

**Authors:** Stéphane Guyot, Lucie Bertheau, Jennifer Dumont, Jean Demarquoy, Hazel Marie Davey, Patrick Gervais

**Affiliations:** a Université Bourgogne Europe, Institut Agro, INRAE, UMR PAM, F-21000, Dijon, France; b Department of Life Sciences, Aberystwyth University, Aberystwyth, United Kingdom

**Keywords:** *Saccharomyces cerevisiae*, Heating kinetics, Transcriptomics, Meta-analysis, Specific signature, Non-specific signature

## Abstract

A central challenge in biology is to distinguish transcriptional responses that recur across heterogeneous stress conditions from those that are specific to particular experimental contexts. Here, using *Saccharomyces cerevisiae* as a model system, we present an integrative transcriptomic framework to address this issue from bulk population-level datasets. We combined 13 carefully curated public transcriptomes with two in-house datasets generated under controlled heat ramp and heat shock conditions. By integrating co-expression network analysis, functional enrichment, and promoter motif discovery, we identified gene modules associated either with recurrent non-specific stress responses or with condition-specific responses linked to gradual heating.

Because bulk transcriptomic measurements capture an integrated signal across cells that may differ in physiological status, including viable, injured, and non-cultivable subpopulations, our conclusions are intentionally framed at the population level rather than at the level of individual cells. Within this framework, heat ramp, in contrast to sudden heat shock, was associated with a broader and more structured population-level transcriptional program enriched in stress-response genes and regulatory motifs linked to increased survival.

Differential transcriptomic responses were associated with ribosome biogenesis, RNA processing, mitochondrial function, and proteostasis, while also showing that stress-responsive gene clusters displayed limited overlap with evolutionarily young genes. To summarize these findings, we propose conceptual models of the transcriptional organizations associated with heat ramp and heat shock and highlight their dependence on heating kinetics. Although developed in yeast, this framework provides a broadly applicable strategy for distinguishing recurrent from condition-specific transcriptomic patterns in heterogeneous stressed populations.

## Highlights

• Bulk meta-transcriptomics separates recurrent and condition-specific responses.
• Heat ramp triggers a broader, more structured program than heat shock.
• WGCNA, GO and motif analyses identify recurrent non-specific modules.
• Heat ramp-specific signatures link ribosome biogenesis and proteostasis.
• Stress-responsive modules show limited overlap with evolutionarily young genes.

## Graphical abstract

**Figure:**
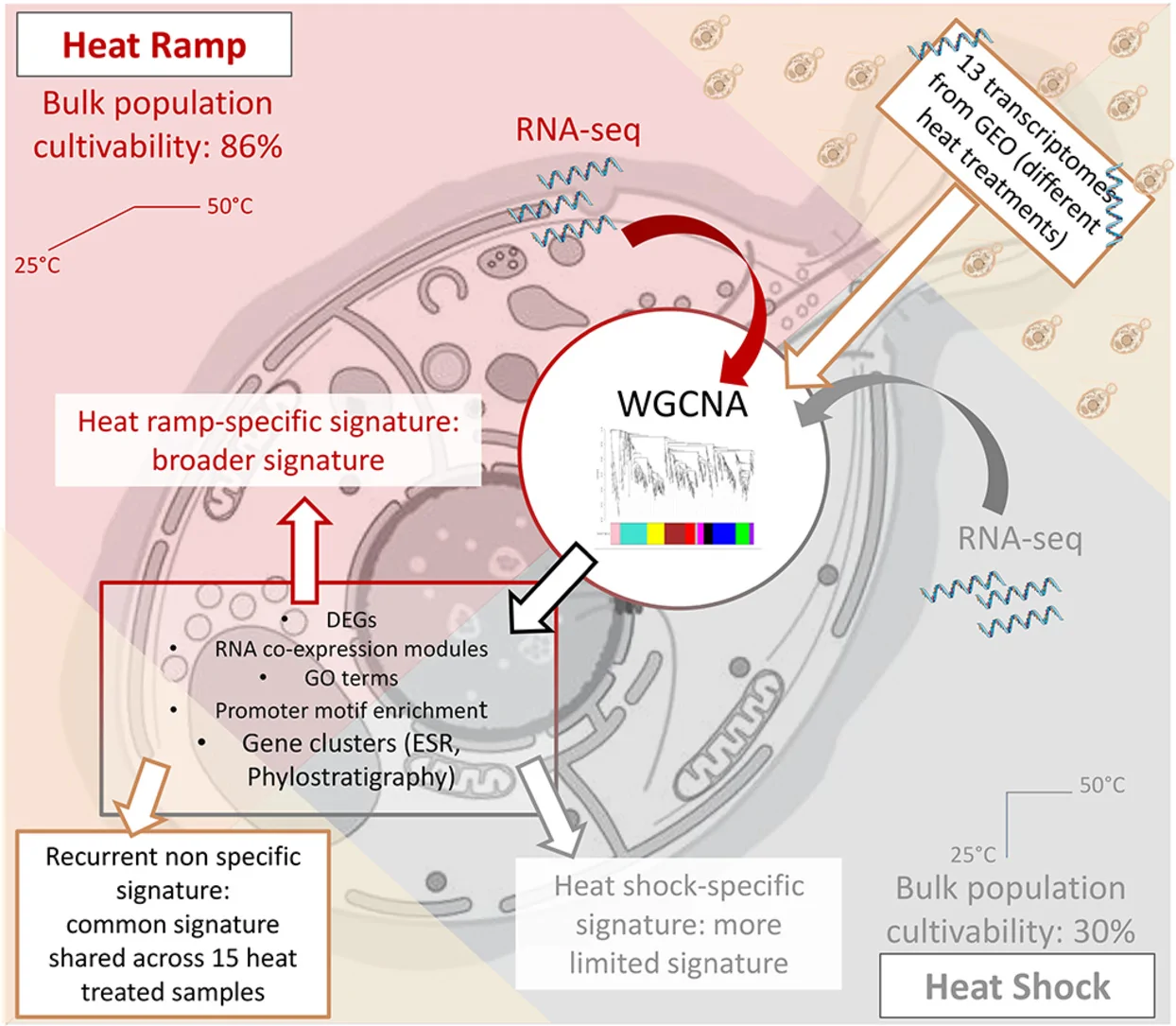

Yeast_cells icon by SwissBioPics https://www.swissbiopics.org/ is licensed under CC-BY 4.0 Unported https://creativecommons.org/licenses/by/4.0/

## 1 Introduction

All living organisms sense and adjust to environmental change to promote continued growth and survival. Although many microbial applications, both in fermentation and in decontamination, were historically developed without detailed knowledge of cellular mechanisms, such knowledge now provides valuable insight for optimizing beneficial microbial systems and improving the control of undesirable microorganisms, notably through a better understanding of resistance mechanisms. In the laboratory, these mechanisms are often studied in isogenic cells, in which adaptation to stress involves coordinated responses across multiple cellular scales. One important component of this adaptation is differential gene expression. Accordingly, stress responses in microbial populations can be studied using high-throughput transcriptomic approaches performed either at the whole-population level (bulk RNA-seq or microarray) or, more recently, at the single-cell level (scRNA-seq) (Gasch et al., 2017).

Bulk transcriptomic approaches provide biologically informative and widely used readouts of stress responses, but they do not resolve cell-to-cell heterogeneity. Under severe or progressive stress, the resulting signal should therefore be interpreted as an integrated population-level output that may combine contributions from subpopulations differing in physiological state, including cells that remain cultivable, cells that are injured, and cells that have lost cultivability. This limitation is not specific to the present study but is inherent to most bulk RNA-seq and microarray studies of stressed populations. Accordingly, throughout this work, we interpret transcriptomic patterns at the population level and do not assume that all cells within a stressed sample share the same molecular state.

A range of bioinformatics tools is available to analyse such transcriptomic datasets, including gene ontology (GO) enrichment analysis, Weighted Gene Co-expression Network Analysis (WGCNA), and transcription factor binding motif enrichment analysis (Ge et al., 2020, 2018; Huang et al., 2009; Langfelder and Horvath, 2008; Mi et al., 2013; Sherman et al., 2022; Thomas et al., 2022). WGCNA can also be combined with analyses of module-trait relationships and hub genes (Langfelder et al., 2013; Niemira et al., 2020). However, the interpretation of transcriptomic datasets depends on the biological question addressed, the structure and heterogeneity of the datasets, and the statistical thresholds used to define meaningful patterns.

The yeast *Saccharomyces cerevisiae* is one of the best studied eukaryotic microorganisms because of its importance in food science, biotechnology, and fundamental cell biology. Since the sequencing of its genome, numerous tools and resources have been developed for functional and systems-level analyses (Engel et al., 2014; Goffeau et al., 1996). Transcriptomic studies have shown that yeast stress responses can be described using several complementary frameworks. One is the Environmental Stress Response (ESR), a broad transcriptional program comprising genes repeatedly induced or repressed across multiple stress conditions, including heat shock, osmotic stress, and oxidative stress. In this framework, the induced ESR (iESR) includes genes involved in stress protection and cellular adaptation, whereas the repressed ESR mainly comprises growth-related functions, particularly ribosome biogenesis (RiBi), ribosomal protein (RP) genes, and other genes linked to RNA metabolism and protein synthesis (Gasch et al., 2000). Another is based on gene age, as phylostratigraphic analyses have suggested that evolutionarily young genes may contribute to stress adaptation in yeasts (Doughty et al., 2020). These frameworks are informative but capture different dimensions of the stress response and are not expected to produce identical gene sets. Importantly, single-cell studies have further shown that the regulation of stress-responsive genes can vary across subpopulations of cells, revealing intrinsic heterogeneity in the yeast stress response (Gasch et al., 2017).

*Saccharomyces cerevisiae* grows optimally between 25 °C and 30 °C, but it is frequently exposed to higher temperatures in both laboratory and industrial settings. Survival under these conditions depends on multiple parameters, including physiological state, temperature, heating kinetics, exposure duration, and medium composition (Caspeta et al., 2014; Gao et al., 2016; Guyot et al., 2015; Hill et al., 2024; López-Malo et al., 1999; Sanchez et al., 1992; Walton and Pringle, 1980). A sudden increase in temperature (heat shock) usually causes marked mortality, whereas a gradual increase to the same final temperature (heat ramp) can induce thermotolerance (Gervais and Martínez de Marañón, 1995; Guyot et al., 2005; Martínez de Marañón et al., 1999; Morozov et al., 1997). This ramp-induced thermotolerance is consistent with the broader framework of acquired stress resistance in yeast (Berry et al., 2011; Berry and Gasch, 2008; Mir et al., 2009; Scholes et al., 2024; Święciło, 2016). Previous studies indicate that these responses are multifactorial and involve membrane properties, proteostasis, stress proteins, trehalose metabolism, and ribonucleoprotein structures (Guyot et al., 2015; Lindquist and Kim, 1996; Ribeiro et al., 1997; Wallace et al., 2015). At the transcriptional level, the yeast heat-stress response is mainly coordinated by the heat shock factor Hsf1, which binds heat shock elements (HSEs), and by the general stress regulators Msn2/Msn4, which act through stress response elements (STREs). These regulatory branches are influenced by upstream signalling pathways including PKA and, in some contexts, Yak1, and are associated with protective proteins involved in thermotolerance and stress adaptation, such as Hsp104 and the oxidative-stress-associated protein Hsp31 (Bankapalli et al., 2015; Verghese et al., 2012). Large-scale approaches have also shown that genes transcriptionally induced by heat are not necessarily the same as those whose deletion leads to strong heat sensitivity (Gibney et al., 2013), and network-based analyses have suggested major rearrangements of cellular functions under heat stress, notably in translation, carbohydrate metabolism, and autophagy (Mihalik and Csermely, 2011). Together, these observations indicate that heat-stress responses cannot be reduced to a single pathway and must instead be analysed as coordinated but condition-dependent population-level transcriptional organizations.

The main objective of this study is to distinguish recurrent non-specific and condition-specific population-level transcriptomic patterns associated with heat stress in *Saccharomyces cerevisiae*. To do so, we integrated public and in-house transcriptomes generated under different thermal conditions, including heat ramp and heat shock, and analysed them using WGCNA, functional enrichment, and promoter motif discovery. We further examined ESR genes and evolutionarily young genes to refine the interpretation of these stress-associated transcriptional patterns. More specifically, we aimed to determine whether a recurrent heat-stress-associated transcriptomic structure could be identified across heterogeneous datasets, and whether gradual heating generated a distinct population-level signature compared with abrupt heat shock.

## 2 Materials and methods

### 2.1 Yeast strain and growth conditions

The haploid strain of *Saccharomyces cerevisiae* BY4742 (*MATα; his3Δ1; leu2Δ0; lys2Δ0; ura3Δ0*) was employed as the wild-type strain.

The yeast cells were cultivated on YPD medium (comprising 1% yeast extract, 2% bacteriological peptone, and 2% d-glucose; Sigma-Aldrich, Saint Quentin Fallavier, France). A single colony, grown on a solidified medium (2% agar), was used to inoculate a 100 mL liquid culture in a 250 mL conical flask. This was shaken at 250 r.p.m. for 48 h at 25 °C, allowing the culture to reach stationary phase.

### 2.2 Thermal treatments

A volume of 1 mL of the yeast culture was transferred to a 1.5 mL microcentrifuge tube and subjected to centrifugation for a period of 5 min at a speed of 2880 x g at a temperature of 25 °C. The cell pellet was resuspended in 1 mL of fresh YPD medium, vortex-mixed, and subsequently subjected to another centrifugation step (5 min, 2880 x *g*, 25 °C). Following the addition of 1 mL of fresh YPD, 100 µL of the resulting suspension was introduced into a 0.5 mL PCR microtube. The sample (100 µL) was subjected to heat treatment using a PCR gradient thermal cycler (MJ Research PTC200, MJ Research, Waltham, USA). The cell suspensions were maintained for a period of five minutes at a temperature of 25 °C, following which they were subjected to either a heat shock or a gradual increase in temperature to 50 °C. The heat shock was applied from 25 °C to 50 °C within 36 s at a rate of approximately 41.6 °C per minute. The heat slope treatment was applied from 25 °C to 50 °C within a period of 50 min, with a rate of 0.5 °C per minute. Subsequently, a 30-minute plateau phase at 50 °C was initiated, following the shock and slope treatments.

### 2.3 RNA sequencing analysis (RNA-Seq)

Yeast total RNA was extracted using hot acid phenol and quantified using Nanodrop2100. RNA quality was checked by capillary electrophoresis on Bioanalyzer 2100 (Agilent Technologies, Santa Clara, USA). All samples had a typical RNA profile with a RIN < 8.

RNA was treated by 1 MBU of Baseline-ZERO™ DNase (ref#DB0715K, Epicentre) for 20 min at 37 °C followed by a phenol/chloroform and chloroform extraction. RNA was precipitated in 3 vol of 96% ethanol (v/v) using 0.3 M sodium acetate and 15 µg of glycoblue. After a wash with 80% ethanol (v/v), the pellet was resuspended in 101 µL of RNase-free H_2_O. After RNA quantification and quality control, 4.5 µg of each DNase I-treated RNA was used for polyA+ isolation using NEBNext Oligo d(T)_25_ Magnetic Beads kit (ref#E7490, NEB) following the manufacturer’s instructions. Enrichment of the polyA+ fraction was checked by capillary electrophoresis on a Bioanalyzer 2100 (Agilent).

1 ng of each rRNA-depleted RNA was converted to a library with the Scriptseq V2 RNA-Seq kit (ref#SSV21106, Illumina) from Illumina using the manufacturer’s instructions.

After 15 cycles of PCR amplification, the libraries were purified using the Agencourt AMPure XP beads (ref#A63880, Beckman Coulter) at a ratio of 0.9x.

Library quality was assessed using a High Sensitivity DNA chip on a Bioanalyzer 2100. All the libraries showed a conventional profile without the presence of dimer adapters.

Library quantification was done using a fluorometer (Qubit 2.0 fluorometer, Invitrogen). Libraries were multiplexed and subjected to high-throughput sequencing using an Illumina HiSeq 1000 instrument with 50 bp single-end read runs and loaded at 12 pM per lane.

Quality of sequencing reads was evaluated by FastQC (https://www.bioinformatics.babraham.ac.uk/projects/fastqc/). Raw sequencing reads were trimmed by Trimmomatic V0.32 (Bolger et al., 2014) with the following parameters: adapters/TruSeq3-SE.fa:2:30:10 LEADING:30 TRAILING:30 SLIDINGWINDOW:4:15 MINLEN:17 AVGQUAL:30.

Trimmed reads were aligned to the SacCer3 assembly of the *S cerevisiae* genome using Bowtie2 (Langmead and Salzberg, 2012) in sensitive-local mode. The *.sam file generated was then sorted and converted to *.bam with subsequent removal of non-mapped reads. Visualization was performed in Integrative Genomics Viewer (IGV) (https://igv.org, 27 August 2024) (Robinson et al., 2023). Raw RNA-seq counts from heat ramp and heat shock experiments were analysed using DESeq2 (Love et al., 2014), which performs normalization and differential expression analysis directly on count data using a negative binomial model. Differential expression was assessed using Wald tests, and p-values were corrected for multiple testing using the Benjamini–Hochberg method.

RStudio software (version 2023.06.0 Build 421) was used to generate the Principal Component Analysis (performed using the FactomMineR (Lê et al., 2008), factoextra (10.32614/CRAN.package.factoextra) and Factoshiny (10.32614/CRAN.package.Factoshiny) packages, **Fig. S1**).

The list of dubious ORFs was retrieved from AllianceMine (https://www.alliancegenome.org/bluegenes/alliancemine/lists) (The Alliance of Genome Resources Consortium, 2024) on January 15, 2026, using the SGD definition of dubious ORFs. Subsequent updates of AllianceMine distinguish a smaller subset of WSO (Without Standard ORF name); however, for consistency across analyses and with previous yeast studies, we retained the original SGD-based definition throughout the manuscript.

### 2.4 Transcriptomic meta-analysis

Transcriptomic datasets were retrieved from the Sequence Read Archive (SRA) or the Gene Expression Omnibus (GEO) in May 2023. Because public datasets did not systematically provide raw gene-level counts (some studies reporting only log_2_ fold-change (log_2_FC) values and adjusted p-values) we applied a harmonized DEG (differentially expressed gene) selection rule across all transcriptomes. Specifically, genes were considered differentially expressed when FDR-adjusted p-values met the same threshold (padj ≤ 0.01), ensuring comparability across heterogeneous input formats and analysis pipelines. log_2_FC values were obtained or calculated depending on data availability. For PRJNA531619 (SRA), log_2_FC values were provided directly. For the BY4741 strain dataset (Mühlhofer et al., 2019), log_2_FC values were kindly provided by the authors. log_2_FC values were calculated for the datasets generated in the present study (heat shock and heat ramp, GSE276309) as well as for GSE182342. For GSE96829, log_2_FC values were computed based on the comparison between ScY01 and ScY strains at 40 °C.

For microarray datasets GSE33276 and GSE15147, raw signal intensities were retrieved and processed as follows. A first normalization step was performed using the *manorm* function in MATLAB R2021b (The MathWorks, Natick, MA, USA) (F532 Median − B532 for Cy3 control and F635 Median − B635 for Cy5 stress). log_2_FC and normalized log_2_FC values were subsequently calculated using the *mairplot* MATLAB function. Probes flagged as “Absent (A)” by the original authors on GEO after the first normalization step were excluded from downstream analyses.

Differential expression analysis for microarray datasets was then conducted using a pairwise comparison strategy implemented with the *mod.t.test* function from the Bioconductor *limma* package (Ritchie et al., 2015), applying moderated *t*-tests to compute p-values and FDR-adjusted p-values. This approach ensures appropriate handling of variance estimation in microarray-based transcriptomes while maintaining consistency with the DEG selection strategy applied across all datasets.

### 2.5 Weighted gene co-expression network analysis (WGCNA)

Prior to gene co-expression network analysis (WGCNA) construction, we assessed the necessity of batch correction to address potential technical variability across datasets originating from different platforms or laboratories. Although batch correction methods such as ComBat are commonly used to reduce platform-specific or laboratory-specific biases in transcriptomic meta-analyses (Jung et al., 2017; Mooney et al., 2013), we chose not to apply them in our final WGCNA pipeline. Batch correction using the ComBat algorithm (Johnson et al., 2007), implemented using the *ComBat()* function from the *sva* package (Leek et al., 2012), was initially tested with the mean.only = TRUE parameter to accommodate batches with a single sample and to correct only for location shifts. Preprocessing steps required by ComBat included filtering genes present in at least 80% of samples and inputting missing values using per-gene means. Preliminary principal component analysis (PCA) using the *prcomp()* function from the *stats* package, and variance homogeneity tests [ANOVA *(aov()*), Levene’s test (*leveneTest()* from the *car* package (doi: 10.32614/CRAN.package.car), and Fligner-Killeen test (fligner.test() (Fligner and Killeen, 1976) from *stats*)] revealed no significant residual effect of sequencing method or laboratory on the first two principal components. Normality of model residuals was assessed using the Shapiro-Wilk test (*shapiro.test()*), and the choice of statistical test (parametric or non-parametric) was based on this criterion. All analyses were performed using R version 2023.06.0 Build 421. Moreover, WGCNA is designed to identify modules of co-expressed genes and is therefore inherently robust to technical noise when biological correlation structures predominate. Applying ComBat required stringent pre-processing, including the removal of genes with missing values in >20% of samples and the exclusion of genes with a log_2_FC equal to zero. However, such values are biologically plausible and frequent in RNA-seq or microarray data. Given the relatively small sample size (n = 15) and the heterogeneous gene coverage across datasets, these filtering steps led to a significant reduction in the number of usable genes and noticeably altered the co-expression network structure. To further assess potential batch effects, we compared PCA results and statistical tests before and after ComBat correction (**Figs S2 and S3**). While the correction effectively removed platform-associated variability, it did so at the cost of gene coverage and biological interpretability. It is also worth noting that some of the variance observed along PC1—particularly with respect to the *Study* factor—may reflect genuine biological differences between studies, such as variation in thermal stress protocols (e.g., stress intensity, duration, or growth phase at exposure), rather than purely technical effects. We therefore prioritized biological signal retention over statistical correction, while transparently documenting these limitations and exploratory analyses.

The WGCNA was conducted using the iDEP 2.01 online tools (http://bioinformatics.sdstate.edu/idep11/, October 2024) (Ge et al., 2018; Langfelder and Horvath, 2008). The gene expression data of the 15 samples referenced in Table 1 and available in **Table S1** (chromosomal coding genes: Poly A+) were analysed with the following parameters: data type: Fold-changes & P-values, soft threshold = 10 (based on the scale independence and mean connectivity graphs, **Fig. S4**), minimum module size = 30, most variable genes included = 1000. Network plots of the top 10 genes per module were drawn with an edge threshold of 0.2. Genes were ranked per WGCNA module colors (**Table S2**). Furthermore, a heatmap was generated based on the WGCNA using the iDEP 2.01 online tools. Regarding the way the data were used, it was noted that the experimental designs were not the same for all studies, as the number of replicates (when available) varied between studies. Therefore, to avoid giving more weight to one part of the data, for each transcriptome the mean of log_2_FC was used instead of log_2_FC for each replicate, which was not always available. In addition, outliers were not removed from the databases to avoid losing information.

**Table 1:** **Biological and methodological metadata associated with the transcriptomes for meta-analysis.** Transcriptomes were based on the expression of Poly A+ mRNA from chromosomal coding genes.(2020; 2010; 2019; 2014; 2015; 2023).

Yeast strain of *S. cerevisiae*	Origin	Ploidy	Heat treatment applied	Initial temperature ( °C)	Heat stress temperature ( °C)	Method	Number of genes	Phenotype induced by the heat treatment	Data analysis performed	Accession no	References
BY4742	Laboratory	Haploid	Heat shock	25	50	Illumina	6666	Low survival	DESeq2 R package⁎⁎	GEO: GSE276309	Present study
			Heat ramp					Heat resistance			
XH7	Industry	Diploid	Propagation at 50 °C	30	50	Illumina	5947	Heat sensitive	DESeq2 R package⁎⁎	GEO: GSE182342	Zhang *et al*., 2023
Z100	Adaptively evolved from XH7						5945	Thermotolerance			
CEN.PK113–7D	Laboratory	Haploid	Chemostat cultivation	30	36	Illumina	5553	Thermotolerance	limma, edgeR and tidyverse R packages	SRA: PRJNA531619†	Doughty *et al*., 2020
BY4741	Laboratory	Haploid	Heat shock	25	37	Microarray	5656	Heat resistance	affy R Package	Data kindly provided by the authors	Mühlhofer *et al*., 2019
ScY01 versus ScY	Industry	Diploid	Propagation at 40 °C	30	40	Illumina	5858	Thermotolerance	limma R package	GEO: GSE96829	Shui et al., 2015††
NFRI 3155	NFRI collection*	Unknown	Heat shock	30	39	Microarray	5757	Heat sensitive	manorm and mairplot Matlab function and limma R package**¶	GEO: GSE33276	Nakamura *et al*., 2014
NFRI 3236	NFRI collection*	Unknown					5757	Thermotolerance			
S288c	Laboratory	Haploid					5858	Intermediate			
DBY8268	Laboratory	Diploid	Heat shock	25	37	Microarray	5952	Heat resistance	manorm and mairplot Matlab function and limma R package**¶	GEO: GSE15147	Eng *et al*., 2010
K9	Sake production	Diploid					7209				
M22	Vineyard	Diploid					5844				
RM11a	Vineyard	Haploid					5688				
YPS163	Oak soil	Diploid					6879				

⁎ NFRI: The Institute of Food Research, NARO, Japan (https://www.naro.go.jp/english/laboratory/nfri/index.html, October 2024).

⁎⁎ Specific data analysis performed for the present study using the raw data collected.

† Data were collected in Supplementary data 1 and 4 of Doughty *et al*., 2020.

†† The data have only been published in GEO (Shui *et al*., 2015 mainly focused on proteomic analysis).

¶ Firstly, the raw data were normalised using the manorm Matlab function (F532 Median-B532 (cy3 Green control) and F635 Median -B635 (cy5 red stress)) (1st normalisation). Subsequently, Log_2_FC and Log_2_FC normalised (2nd normalisation) were calculated using mairplot Matlab function. The data that had been subjected to the first round of normalisation and displayed an 'Absent (A)' flag by the authors on GEO were not included in the subsequent calculations of Log_2_FC and Log_2_FC normalised. Secondly, a pairwised calculation of Log_2_FC was performed, utilising the mod.t.test function to determine the pval and padj based on the Bioconductor limma R package (moderated *t*-test for microarray).

### 2.6 Curation and coding of traits for WGCNA module colors–trait correlations (Table S3)

Trait information was curated from GEO metadata and the associated publications and summarized in Table 1, including the temperature kinetics and exposure duration. Three traits were used for module–trait correlations: viability-related outcome, strain origin, and ploidy. “Viability” was coded as an ordinal proxy reflecting the expected outcome of the heat treatment and/or author-reported endpoints: 1 (low viability), 5 (intermediate), and 10 (resistance/adaptation). When explicit phenotyping was not reported, the score was assigned conservatively based on the study aim and treatment severity (sublethal/adaptive vs lethal), and should therefore be interpreted as a qualitative proxy rather than a measured phenotype. “Origin” was coded as 1 (industrial) or 2 (collection/laboratory). “Ploidy” was coded as 1 (haploid), 2 (diploid), or 0 (unknown).

### 2.7 Gene ontology analysis

A GO Biological Process analysis was conducted using the online tool Panther 19.0 (January 29, 2026, https://pantherdb.org/) (Mi et al., 2021, 2019; Mi and Thomas, 2009; Thomas et al., 2022). The analysis was performed after the identification of the dubious ORFs, which were then removed as explained in the 'RNA sequencing analysis (RNA-Seq)' paragraph. A PANTHER Overrepresentation Test was conducted utilising the GO Ontology database (from GO database released 2025–10–05, DOI: 10.XXXX/zenodo.XXXXXXXX). The uploaded reference gene list is as follows: *Saccharomyces cerevisiae*. The test was performed on the reference gene list for *Saccharomyces cerevisiae*. The dataset used was GO Biological Process complete (GO BP), and the test was conducted using Fisher's exact test with false discovery rate (FDR) correction. An FDR of 0.01 was employed as the cutoff threshold for the selection of significant GO BP terms.

### 2.8 Enrichment motif in promoter sequences

The enrichment motifs in the promoter sequences were identified using ShinyGO 0.77 (http://bioinformatics.sdstate.edu/go77/), a graphical tool for gene enrichment analysis (Ge et al., 2020). The tool was employed in August 2023 with comparable data sets to those generated for verification in November 2024. Notably, ShinyGO 0.77 was based on Ensembl Release 104, archived on 1 February 2026. Subsequent versions (ShinyGO 0.80 and 0.85) of the software do not permit users to perform such an analysis. Any dubious open reading frames (ORFs) were excluded from the gene lists before analysis. The parameters were set as follows: false discovery rate (FDR) = 0.01, # pathways to show = 20, Pathway size: Min = 10 and Max = 20, Remove redundancy: yes, Abbreviate pathways: yes, Upstream 600 bp as a promoter, Use pathway database for gene counts: yes, Using selected species *Saccharomyces cerevisiae*. Only those motifs with a FDR between 1E-5 and 1E-20 were considered to be significantly enriched. Those displaying a FDR of 0 were not considered, as this may indicate an issue with the method, such as overfitting or bias. Overfitting refers to a situation where the enrichment tool or input data may be biased, leading to an artificially inflated significance. Similarly, bias may result from a small sample size, whereby statistical tests may not behave as expected. Incorrect p-value correction may also result in an FDR of 0, whereby the adjusted p-value is exceedingly small but not exactly 0. From a statistical standpoint, FDR values should be exceedingly small (e.g., between 1E-5 and 1E-20) for highly significant results, though they should not be exactly 0.

The promoter motif enrichment was generated using the motif logo tool from the SRplot graphical interface (February 2026) (Tang et al., 2023).

### 2.9 Protein-protein interactions (PPIs) network

Protein–protein interaction analyses were performed using gene sets derived from WGCNA modules. Dubious ORFs were retained at this stage, as they are not associated with annotated protein interactions and are therefore ignored by PPI databases, preventing the introduction of annotation-driven bias while preserving the integrity of module-derived gene sets. Protein-protein interaction networks were analysed using the Multiple proteins option from STRING, a database and tool used to predict protein-protein interactions (PPIs), based on both experimental data and computational predictions (November 2024, v12.0, https://string-db.org) (Szklarczyk et al., 2023). The highest confidence interaction threshold (score ≥ 0.9) was applied, incorporating both experimental and database evidence. Furthermore, a tabular matrix was exported and imported into Cytoscape 3.10.3, which was used to generate the graphical network (November 2024, https://cytoscape.org/) (Franz et al., 2023).

### 2.10 Statistical analysis of HS vs HR comparisons by RNA class

All statistical analyses were performed in RStudio (version 2023.06.0 Build 421) using R. Differential expression values (log2 fold-change; log_2_FC) were compared between the heat shock (HS) and heat ramp (HR) conditions separately for each RNA biotype (mtRNA, ncRNA, snoRNA, tRNA, and snRNA). For each biotype, log_2_FC values were extracted and assigned to one of the two experimental groups (HS or HR), and a two-group comparison was conducted. To select an appropriate test while accounting for model assumptions, we implemented an automatic decision strategy based on (i) normality of ANOVA residuals and (ii) homogeneity of variances. Specifically, an ANOVA model (*aov(), package stats*, log_2_FC ∼ Stress) was fitted for each RNA class and the Shapiro–Wilk test (*shapiro.test(), package stats*) was applied to the model residuals (using *residuals()*). When residuals were consistent with normality (Shapiro–Wilk *p* > 0.05), homogeneity of variances was evaluated using Bartlett’s test (*bartlett.test(), package stats*). If both assumptions were met (Shapiro–Wilk *p* > 0.05 and Bartlett *p* > 0.05), a two-sample Student’s *t*-test (*t.test(…,* var.*equal = TRUE, package stats*, two-sided, α = 0.05) assuming equal variances was applied to compare HS and HR. If either assumption was violated (residual normality and/or variance homogeneity), the comparison was performed using the Wilcoxon rank-sum test (*wilcox.test(…, exact = FALSE, package stats*, Mann–Whitney U) (two-sided, α = 0.05). This approach ensured that HS vs HR comparisons were not systematically analyzed using Student’s *t*-test when parametric assumptions were not satisfied.

Using this strategy, Student’s *t*-tests were applied for mtRNA (Shapiro residuals *p* = 0.9825; Bartlett *p* = 0.407; *t*-test *p* = 0.0603), ncRNA (Shapiro residuals *p* = 0.177; Bartlett *p* = 0.299; *t*-test *p* = 0.699), and snRNA (Shapiro residuals *p* = 0.328; Bartlett *p* = 0.070; *t*-test *p* = 0.00826). In contrast, non-parametric Wilcoxon rank-sum tests were used for snoRNA (Shapiro residuals *p* = 0.01015 and Bartlett *p* = 0.0187; Wilcoxon *p* = 4.14 × 10⁻⁵) and tRNA (Shapiro residuals *p* = 1.53 × 10⁻⁷; Bartlett *p* = 0.94; Wilcoxon *p* = 0.00332), reflecting violations of at least one parametric assumption.

### 2.11 Data visualisation

Violin plots and upset plots were constructed using the SRplot user graphical interface (January-February 2026) to illustrate the relationship between Gene Ontology (GO) Biological Process (BP) terms and weighted gene co-expression network analysis (WGCNA) module colors. (Tang et al., 2023). Venn diagrams were constructed using jvenn user graphical interface (https://www.bioinformatics.com.cn/static/others/jvenn_en/index.html) (Bardou et al., 2014).

## 3 Results

### 3.1 Changes in heating kinetics induce major changes in yeast transcriptome based on the expression of chromosomal coding genes

Stationary-phase cultures were deliberately selected for the present comparison. This choice maintains continuity with our previous physiological studies on the effects of heating kinetics and reflects the food-science context of this work, in which thermal processes frequently act on dense and physiologically heterogeneous microbial populations that are not necessarily undergoing exponential growth. Stationary-phase cells also display greater intrinsic thermal resistance than exponentially growing cells, allowing the effects of heat ramp and severe heat shock to be compared while limiting the risk of an excessive loss of cultivability. In addition, their high and reproducible cell concentration provided sufficient biomass for RNA extraction. However, because stationary-phase cells already exhibit physiological and transcriptional adaptations associated with nutrient limitation and stress resistance, the profiles described below should be interpreted as population-level heat-stress responses superimposed on a pre-adapted stationary-phase state.

RNA-seq analysis was performed on yeast exposed to two different heat treatments associated with distinct levels of loss of cultivability while still allowing transcriptional activity to be captured. In this study, a heat ramp (0.5 °C min⁻¹) and a heat shock (within 30 s), both from 25 °C to 50 °C, were applied in the presence of nutrients, followed by a 30 min incubation at 50 °C before recovery at 25 °C. Both thermal treatments were designed to reach the same severe endpoint of 50 °C and were followed by the same 30-min holding period. This design allowed the effect of heating kinetics to be examined while maintaining the final temperature and exposure duration constant. The heat-shock condition should therefore be considered a severe heat treatment rather than a conventional mild or sublethal heat shock. Indeed, the ramp and shock treatments reduced cell cultivability by no >14% (equivalent to a −0.06 log10 change) and 70% (−0.45 log10 change), respectively (Ragon et al., 2023). RNA extractions for the present transcriptomic analyses were performed by the same research team, in the same laboratory, and during the same experimental period as the cultivability measurements previously reported in Ragon et al. (2023). Although transcriptomic analyses and cultivability measurements were conducted on independent biological cultures, all experimental parameters were strictly identical between the two studies, including growth conditions, heat-treatment kinetics, final temperature (50 °C), duration of exposure, and sampling procedures. This ensures direct comparability between physiological and transcriptomic responses at the population level.

Importantly, the transcriptomes analysed here were obtained from bulk populations and therefore reflect integrated molecular outputs rather than homogeneous single-cell states. Under both heat ramp and heat shock conditions, and especially under the more severe heat shock endpoint, the measured RNA signal may arise from a mixture of subpopulations that differ in physiological status, including cultivable, injured, and non-cultivable cells. Consequently, the transcriptomic profiles reported here should be interpreted as population-level signatures of the stressed samples collected at the selected endpoint, including responses activated during the course of the treatment before irreversible loss of cultivability. In this context, the heat shock transcriptome should not be interpreted as a snapshot of the surviving fraction alone, whereas the heat ramp condition reflects a distinct physiological trajectory associated with higher overall cultivability.

**Fig. 1:**
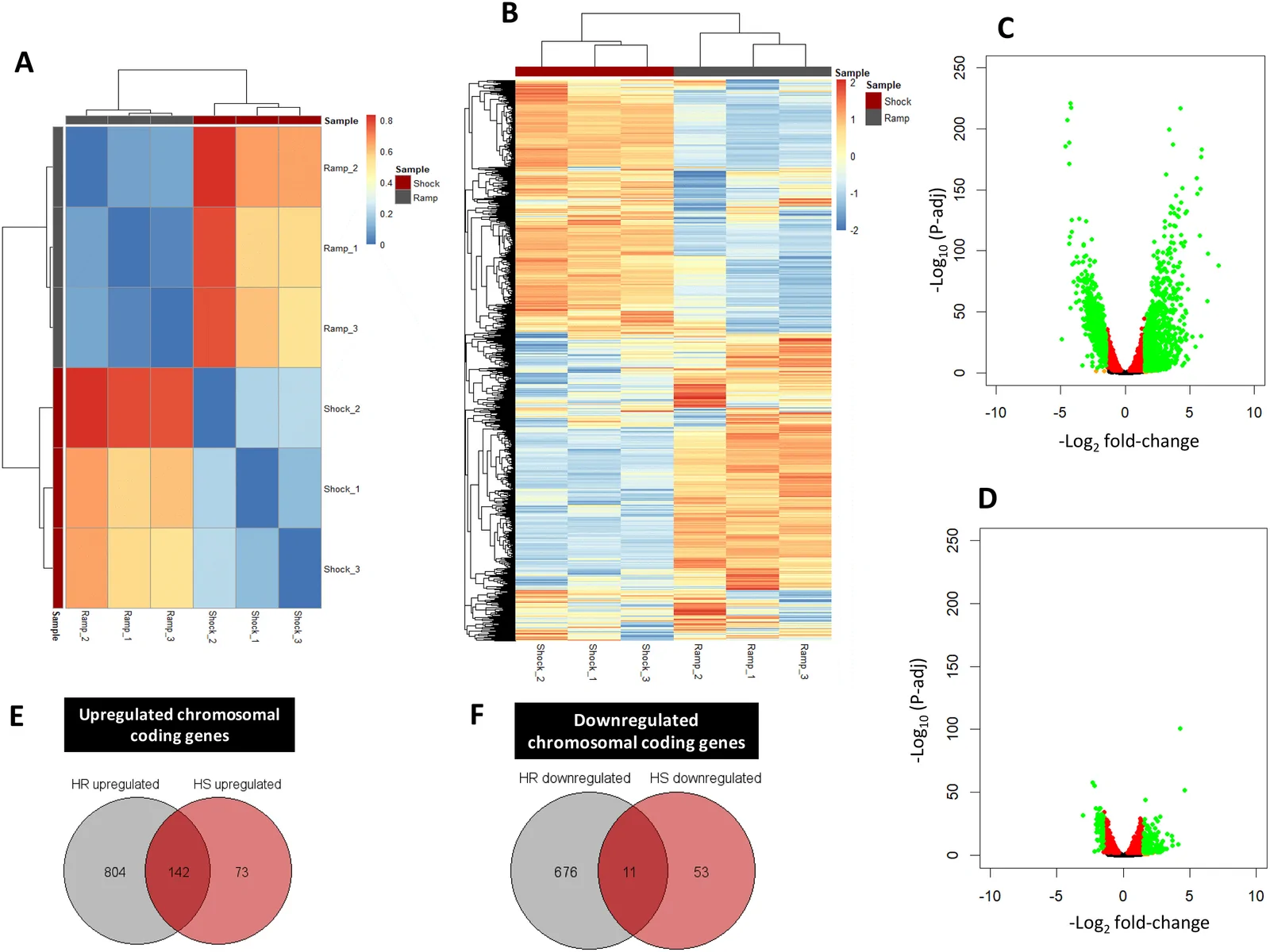
Differentially expressed chromosomal coding gene analysis of *S. cerevisiae* BY4742 after a ramp and a shock from 25 °C to 50 °C followed by a 30 min holding time at 50 °C in YPD medium. A-F: only Poly A+ mRNAs from chromosomal coding genes were considered. Both A: sample-to-sample correlation and B: Hierarchical clustering heatmap comparing heat shock and heat ramp transcriptomes. Rows represent genes and columns represent biological replicates (Shock_1–Shock_3; Ramp_1–Ramp_3). Heatmap values correspond to log_2_FC relative to unstressed controls (color scale). Both genes (rows) and samples (columns) were hierarchically clustered; the top annotation indicates the stress condition (Shock vs Ramp). The heatmap is shown to visualize global expression-pattern differences between heat shock and heat ramp and replicate consistency; functional interpretation of the opposing gene patterns is provided by GO BP enrichment analyses of the corresponding DEG sets (Fig. 4; Tables S12–S14; dubious ORFs excluded) . Volcano plots of gene expression change after C: the ramp and D: the shock, genes with a padj () ≤ 0.01, () absolute value of Log_2_FC ≥ 1.5, () both. Venn diagrams comparing E: upregulated and F: downregulated genes after a ramp (HR) and a shock (HS), only genes with both a padj ≤ 0.01 and a log_2_ fold-change ≥ 1.5 or ≤ −1.5 for upregulated and downregulated ones respectively were considered. Three independent repetitions per condition were performed.

Polyadenylated mRNAs from chromosomal coding genes were first analysed using sample-to-sample correlation (Fig. 1**A**), hierarchical clustering (**Fig. 1B**; heatmap of log_2_ fold-change (log_2_FC) relative to unstressed controls showing replicate consistency and global separation between heat shock and heat ramp), and volcano plots (Fig. 1**C–D**), based on a stringent differential expression threshold (padj ≤ 0.01 and absolute log_2_FC ≥ 1.5). This stringent selection was chosen to highlight robust transcriptional changes and to prioritize high-confidence candidates for downstream interpretation.

These analyses showed that heat ramp and heat shock generated clearly distinct population-level transcriptional outputs. The gene expression profile induced by the heat ramp differed markedly from that observed after heat shock, whereas heat shock transcriptomes showed only partial, but not complete, separation from the unstressed condition (**Fig. S1**). Among the 6666 chromosomal coding genes analysed, 804 and 676 genes were specifically upregulated and downregulated, respectively, after the heat ramp (Fig. 1**E–F; Tables S3 and S4**). In contrast, approximately tenfold fewer genes were differentially expressed following heat shock under the same stringent criteria (**Tables S3 and S5**), indicating that slower heating kinetics are associated with a broader transcriptional response. Overall, slightly >10% of yeast genes exhibited differential expression during the heat ramp, whereas a substantially smaller fraction was detected during heat shock under these stringent conditions.

Despite these major differences, a subset of 142 genes was consistently upregulated and 11 genes downregulated under both heating regimes (Fig. 1**E–F; Table S3**), suggesting that the regulation of these 153 genes depends primarily on the final temperature and/or the duration of exposure at 50 °C rather than on heating kinetics alone.

To further characterize the nature of these transcriptional responses, we quantified the proportion of dubious and non-dubious ORFs within the different DEG (differentially expressed gene) sets (**Table S3**). This analysis revealed a condition-dependent enrichment of dubious ORFs, particularly under heat shock when using stringent selection criteria. Indeed, DEGs from the heat ramp and heat shock conditions included, respectively, about 15% and 41% of dubious ORFs (**Table S3**).

Finally, sensitivity analyses were performed to evaluate the impact of DEG selection thresholds on both Heat Ramp versus Control and Heat Shock versus Control comparisons. The number of DEGs was analysed as a function of the absolute log_2_FC, with two curves generated for each comparison using padj ≤ 0.01 and padj ≤ 0.05 (**Tables S4 and S5**). A complementary DEG list based on the less stringent criteria padj ≤ 0.05 and absolute log_2_FC ≥ 1 is also provided (**Table S6**). These analyses show that the transcriptional response induced by the heat ramp is robust across thresholds, whereas the number of genes detected after heat shock depends much more strongly on the applied stringency. This supports the interpretation that the limited number of DEGs observed under heat shock with the stringent threshold reflects an analytical choice rather than an intrinsic limitation of the dataset.

Taken together, these results indicate that heating kinetics strongly shape transcriptomic organization at the population level, with heat ramp eliciting a broader and more structured response than heat shock under the endpoint conditions analysed here.

### 3.2 Identification of a yeast sub-transcriptome common to all heat treatments

To determine whether a recurrent transcriptomic signature could be identified across heterogeneous heat treatments in *S. cerevisiae*, a transcriptomic meta-analysis was conducted using 13 previously published transcriptomes together with the two additional transcriptomes generated in the present study under heat ramp and heat shock conditions. All transcriptomes focused on protein-coding genes, yielding a combined dataset of 7213 genes (**Table S1**). Because these studies differed in experimental design and analytical platform (RNA-seq or microarray), not all genes were consistently measured across all transcriptomes. Rather than assuming that such heterogeneous studies are directly comparable at the level of individual gene fold changes, we used this diversity intentionally to identify co-expression patterns that recur across independent heat-stress experiments. The transcriptomes considered covered a range of heat-treatment modalities (Table 1), spanning conditions expected to result in adaptation/resistance, intermediate outcomes, or reduced viability depending on the study design and endpoints.

**Fig. 2:**
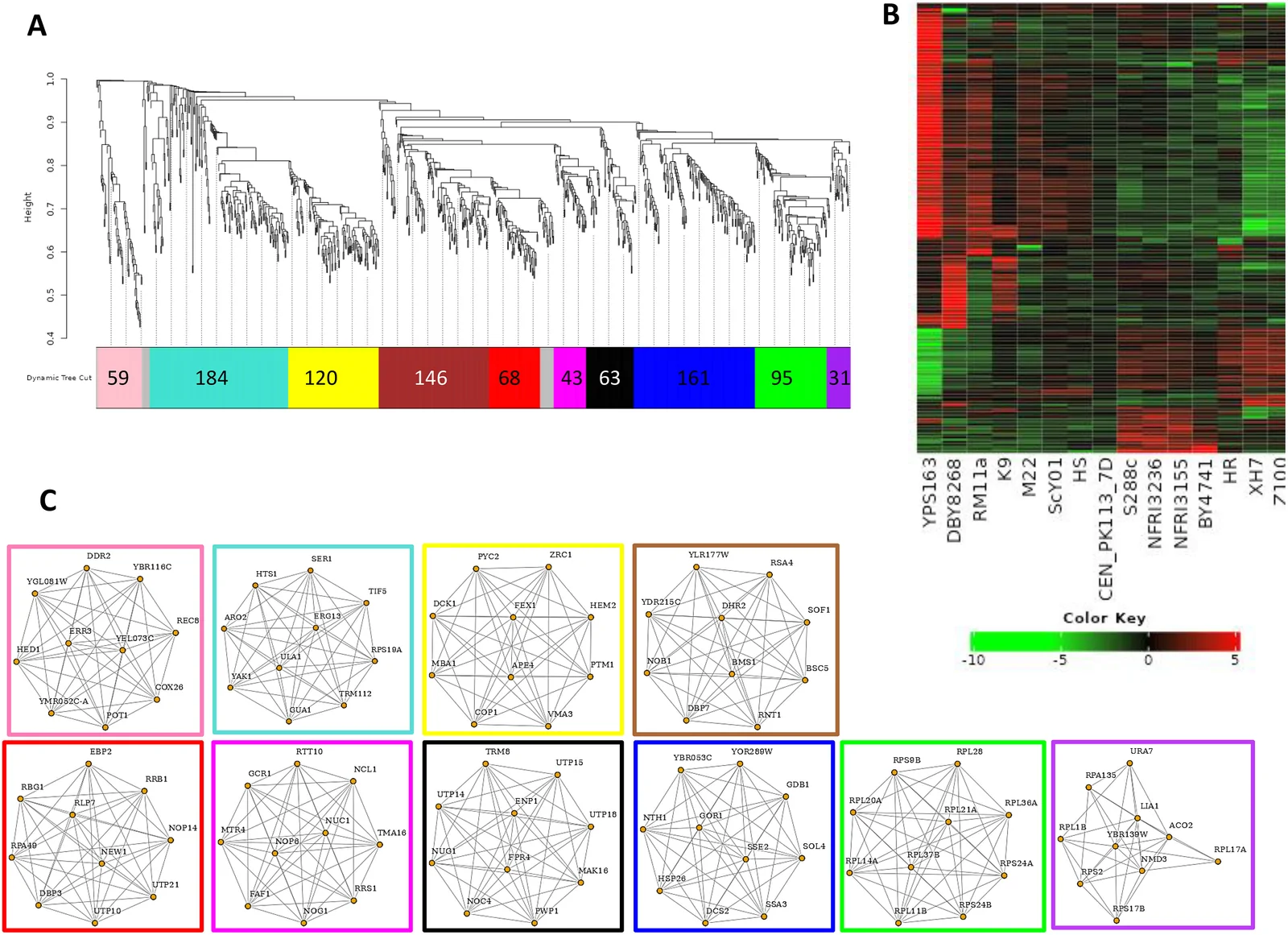
**Weighted Gene Co-expression Network Analysis (WGCNA) in heat treated strains of *Saccharomyces cerevisiae*.** Transcriptomes were based on the expression of Poly A+ mRNA from chromosomal coding genes. Biological and methodological metadata are presented in Table 1. **A:** Gene dendogram and module colors with number of genes per module. **B:** Overview heatmap illustrating the diversity of transcriptomic responses included in the meta-analysis. Columns correspond to individual transcriptome datasets (including GEO studies); rows correspond to genes (y-axis). The heatmap displays the 1000 most variable protein-coding genes (PolyA+) retained for WGCNA (iDEP parameter). Gene identifiers are not displayed because this panel is for overview only and labels would be unreadable at this scale. Values are log_2_FC for heat-related conditions relative to the corresponding unstressed control within each study. The color scale represents log_2_FC (green: down-regulation; red: up-regulation; range −10 to +5). For time-course studies, the time point retained is indicated in Table 1. The heatmap was generated with iDEP (see Materials and Methods) and is shown for overview purposes only (no clustering-based inference is drawn from this panel). **C:** Network representation of the 10 most connected genes (top candidates) within each WGCNA module, identified using iDEP (WGCNA implementation) and ranked according to their intramodular connectivity. Nodes represent genes and edges represent strong expression similarity (co-expression) across the transcriptomes included in the analysis using an edge threshold of 0.2. This panel illustrates how modules are built from highly co-expressed genes rather than differential expression and visualizes the gene-level co-expression architecture underlying each module; it is not intended as an independent functional-enrichment analysis.

Weighted Gene Co-expression Network Analysis (WGCNA) identified ten co-expression modules based on similarity in expression profiles across the 15 transcriptomes (Fig. 2**A**). The grey module did not correspond to any coherent co-expression cluster, indicating that the associated genes did not exhibit a sufficiently consistent pattern across the datasets. In total, 970 genes among the 7213 initially considered were distributed across the ten modules, with module sizes ranging from 31 to 146 genes (Fig. 2**A, Table S2**). Despite the limited number of transcriptomes included in the analysis (n = 15), the WGCNA approach was effective in identifying recurrent gene clusters. The overview heatmap further illustrated the marked heterogeneity of log_2_FC responses across the transcriptomes included in the meta-analysis, thereby highlighting why direct cross-study comparison of gene-level responses is not straightforward (Fig. 2**B**). Accordingly, the modules identified here should be interpreted as recurrent population-level transcriptional patterns recovered across heterogeneous heat-stress experiments, rather than as evidence for identical cellular states across all cells or studies.

To illustrate the co-expression structure underlying module definition, the top ten genes with the highest intramodular connectivity within each module were identified (Fig. 2**C**). This commonly used WGCNA network representation highlights the most strongly co-expressed genes within each module and complements the dendrogram and module assignment shown in Fig. 2**A**. The genes described below are illustrative examples drawn from several modules rather than a specific focus on trehalose metabolism. Consistent with later functional annotation, hub candidates in the ‘Trehalose and carbohydrate metabolism’ module included trehalose turnover genes such as *NTH1*, whereas translation- and ribosome-related modules contained genes such as *RPL14A* and *RPS9B* together with factors involved in ribosome biogenesis and pre-rRNA processing, including *DHR2, FAF1, NOB1, RLP7*, and *PWP1*. Since one objective of WGCNA is to identify modules potentially associated with phenotypic variation, Spearman correlations were calculated between module eigengenes (defined as the first principal component summarizing the expression profiles of all genes assigned to a given module) and the three curated traits (viability, origin, and ploidy). However, no significant module–trait correlation was detected, most likely because phenotypic information extracted from GEO records and the literature remained incomplete and heterogeneous (**Table S7**).

**Fig. 3:**
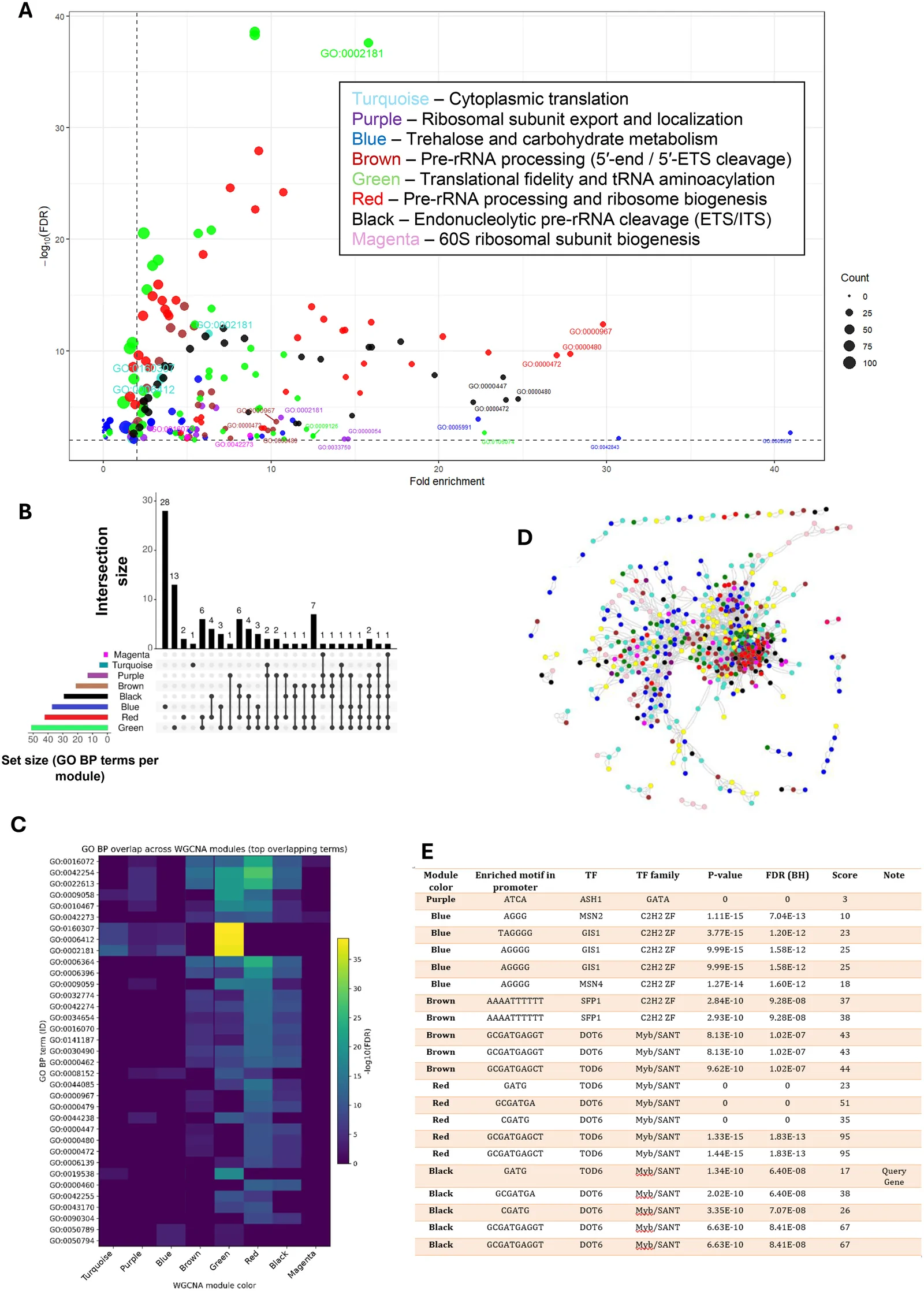
**Characteristics of the module color as identified by the WGCNA.** Transcriptomes were based on the expression of Poly A+ mRNA from chromosomal coding genes. **A:** Bubble plot illustrating Gene Ontology (GO) Biological Processes (BP) fold-enrichment for each module color (excluding dubious ORFs). The size of dots represents the number of counts in each module color. The colors correspond to the color of each module. The inset shows the functional name of each module color, which is based on its most enriched GO BP terms (see Table S8 for the complete list of GO BP terms). The graph shows the three most enriched GO BP terms for each module color. **B:** Upset plot demonstrating the number of shared GO BP terms (excluding dubious ORFs) between module colors. **C**: Heatmap of overlapping GO Biological Process (BP) enrichments across WGCNA modules (excluding dubious ORFs). Rows correspond to GO BP terms (GO accession numbers) and columns to WGCNA module colors. The heatmap is based on -log_10_(FDR) values from the GO BP enrichment analysis. Only GO BP terms enriched in at least two modules are displayed, and for readability the plot is restricted to the top 35 overlapping terms. 0 indicates that the GO term was not reported as significantly enriched for that module in the selected list. **D:** STRING protein functional association network across WGCNA modules. Nodes represent yeast proteins encoded by genes assigned to WGCNA modules (node color = module color). Edges represent STRING-supported functional associations (physical and/or indirect), supported by multiple evidence channels (genomic context, homology transfer, co-expression, experiments, curated databases, and text mining). Edge thickness is proportional to the STRING combined confidence score (0–1). The complete interaction list and evidence-channel scores are provided in Table S9. **E:** Module-specific promoter motif enrichment (up to five motifs per WGCNA module). Promoter motif enrichment was performed separately for each WGCNA module using module-specific DEG lists (dubious ORFs excluded). For each module color, the figure reports up to five enriched promoter motifs (and their associated predicted transcription factors, TFs), selected as the motifs with the lowest Benjamini–Hochberg FDR within that module. Only motifs with FDR between 1 × 10⁻⁵ and 1 × 10⁻²⁰ are shown; cases where FDR = 0 were kept as reported by the software output (reflecting numerical underflow for extremely small values). The ‘Score’ column corresponds to the internal ranking metric returned by the motif-enrichment tool and is reported for completeness; motif selection was based on FDR. Because the motif library contains closely related variants (e.g., different lengths or flanking nucleotides), the pipeline can report multiple near-duplicate motif entries with distinct enrichment statistics and FDR values. The complete per-module promoter motif enrichment output (including all motif/TF hits and associated statistics) is provided in Table S10.

To improve biological interpretability, a Gene Ontology (GO) Biological Process analysis was then performed after exclusion of dubious ORFs, and each WGCNA module was assigned a functional name based on its most representative enriched GO Biological Process terms (Fig. 3**A, Table S8**). Most GO BP terms were shared across at least two modules, indicating that the recurrent heat-stress-associated co-expression structure is not functionally partitioned into fully independent blocks. Nevertheless, some modules retained more distinctive functional profiles. In particular, the ‘Trehalose and carbohydrate metabolism’ (Blue), ‘Translational fidelity and tRNA aminoacylation’ (Green), ‘Pre-rRNA processing and ribosome biogenesis’ (Red), and ‘Cytoplasmic translation’ (Turquoise) modules displayed 28, 13, 2, and 1 module-specific GO terms, respectively (Fig. 3**B**). No GO BP term was associated with the Pink or Yellow modules. The overlap heatmap showed that shared enrichments grouped into coherent biological blocks, notably around translation, ribosome biogenesis, and RNA processing (Fig. 3**C**). For example, translation- and protein synthesis-related terms were recurrent across the Turquoise, Purple, Blue, and Green modules, whereas rRNA processing and ribosome biogenesis terms were repeatedly enriched across modules involved in ribosome maturation.

Consistent with this functional overlap, STRING analysis revealed a dense network of functional associations among proteins encoded by genes assigned to different WGCNA modules (Fig. 3**D; Table S9**), indicating that these modules are not functionally isolated. Although informative, this network-level result should be viewed as a refinement of the functional interpretation rather than as the primary evidence for module definition.

To add a regulatory layer to this recurrent co-expression structure, promoter motif enrichment was performed separately for each module using module-specific DEG lists after exclusion of dubious ORFs. This analysis identified multiple enriched promoter motifs associated with predicted transcription factors across modules (Fig. 3**E; Table S10**). For clarity, Fig. 3**E** summarizes up to five motifs per module, selected as the lowest FDR hits within each module and restricted to motifs with FDR values between 1 × 10⁻⁵ and 1 × 10⁻²⁰, whereas the full output is provided in **Table S10**. Overall, these results suggest that the transcriptional programs captured by the recurrent WGCNA modules are controlled by multifactorial regulation rather than by a single dominant transcription factor. Importantly, promoter motif enrichment does not measure transcription factor activity directly; instead, it indicates that specific cis-regulatory motifs are statistically over-represented in the promoters of the genes considered. Therefore, the absence or weak enrichment of a canonical motif such as that of Hsf1 in a given module should be interpreted as a lack of significant motif over-representation under the selected thresholds rather than as evidence that the heat response itself was not captured.

Taken together, these results indicate that heterogeneous heat-stress transcriptomes in *S. cerevisiae* share recurrent non-specific co-expression modules with coherent functional and regulatory signatures. These modules define a common population-level heat-stress-associated sub-transcriptome that can be distinguished from the condition-specific signatures analysed in the following section.

### 3.3 Toward identifying a yeast sub-transcriptome specific to heat ramp and heat shock

The next objective was to identify transcriptomic signatures that were specific to heat ramp and heat shock in *S. cerevisiae* BY4742, beyond the recurrent non-specific patterns captured by the cross-study WGCNA analysis. To address this question, we compared condition-specific DEGs, Gene Ontology (GO) Biological Process (BP) enrichments, and promoter motif enrichment profiles for the two heating regimes, and then related these signatures to the recurrent WGCNA-derived modules.

**Fig. 4:**
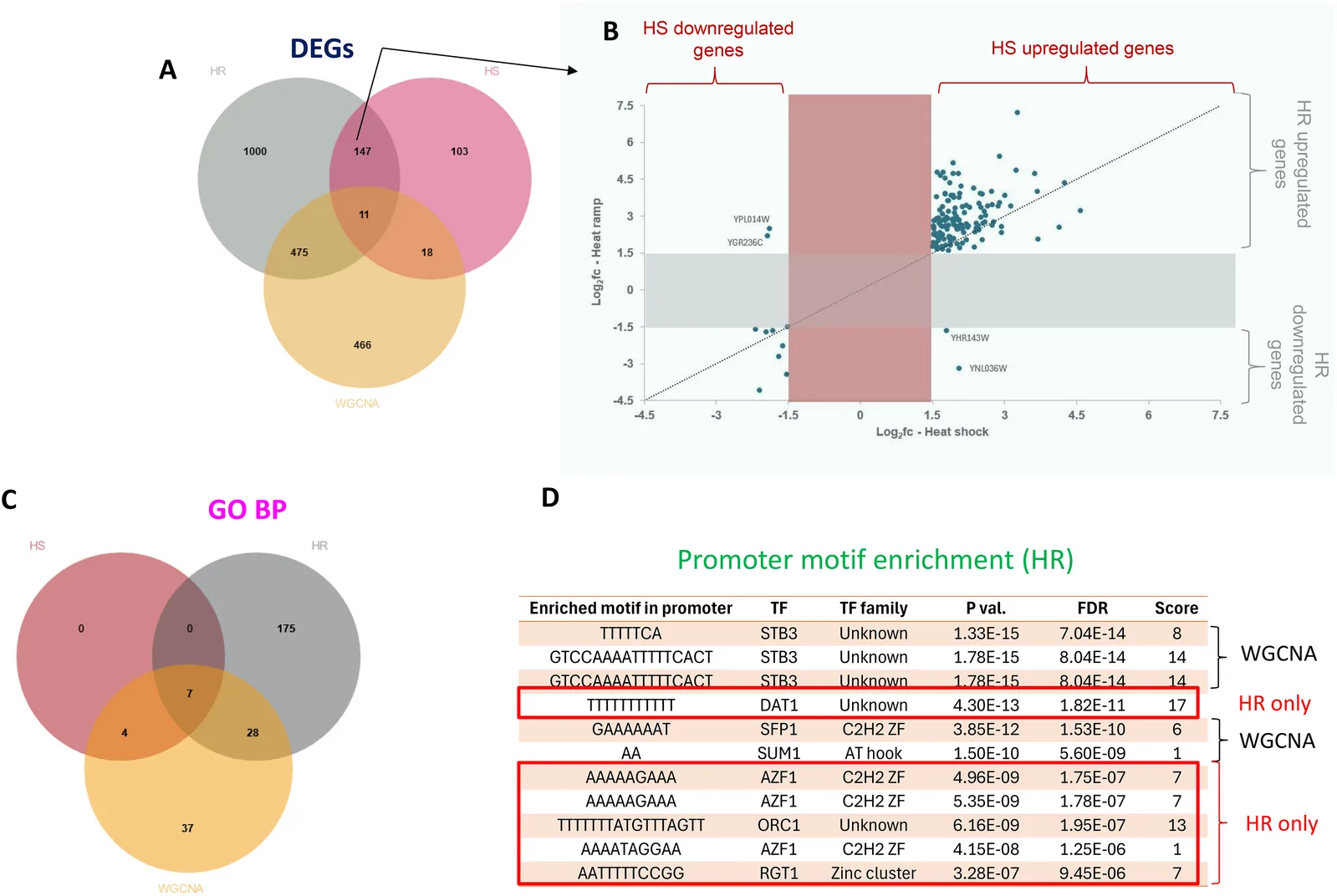
Specific transcriptomic signatures as a function of the heating kinetics in *S. cerevisiae* BY4742. **A:** Venn diagram comparing DEGs displayed in ramp, shock and WGCNA. **B:** log_2_FC of 147 DEGs shared between the ramp and the shock. **C:** Venn diagram comparing GO BP terms (excluding dubious ORFs) connected to the ramp, the shock and WGCNA. **D:** Promoter motif enrichment based on differentially expressed genes (DEGs, excluding dubious ORFs) from yeasts exposed to the heat ramp. Only motifs with FDR between 1 × 10⁻⁵ and 1 × 10⁻²⁰ are shown; cases where FDR = 0 were kept as reported by the software output (reflecting numerical underflow for extremely small values). The ‘Score’ column corresponds to the internal ranking metric returned by the motif-enrichment tool and is reported for completeness; motif selection was based on FDR. Because the motif library contains closely related variants (e.g., different lengths or flanking nucleotides), the pipeline can report multiple near-duplicate motif entries with distinct enrichment statistics and FDR values. Promoter motifs enriched only during the ramp (HR only) and those enriched both during the ramp and in module colors from WGCNA (WGCNA) are identified. (**HR:** Heat Ramp, **HS:** Heat Shock).

Analysis of DEG signatures first revealed a marked asymmetry between the two conditions. Among the 1633 genes identified as differentially expressed across heat ramp and heat shock (including dubious ORFs), 1000 were specific to the heat ramp, whereas only 103 were specific to the heat shock (Fig. 4**A, Table S11**). These genes were not represented within the WGCNA modules and therefore corresponded to condition-specific components rather than to the recurrent non-specific heat-stress signature identified across heterogeneous datasets. Shared DEGs also showed substantial quantitative divergence between the two heating conditions. Among the genes common to heat ramp and heat shock, only four displayed opposite directions of regulation: *YPL014W* (*CIP1*) and *YGR236C* (*SPG1*) were downregulated in heat shock but upregulated in heat ramp, whereas *YHR143W* (*DSE2*) and *YNL036W* (*NCE103*) showed the opposite pattern (Fig. 4**B**). The apparent discrepancy between the number of common DEGs in Figs. 1**E–F** (142 + 11 = 153) and Fig. 4**A** (147 + 11 = 158) results from the fact that, in Figs. 1**E–F**, upregulated and downregulated genes were analysed separately, whereas in Fig. 4**A** they were pooled. Overall, these results indicate that heat ramp and heat shock share only a limited common DEG core and that the vast majority of the condition-specific response is associated with heat ramp.

Functional enrichment analysis reinforced this asymmetry. The heat ramp-induced thermotolerance condition was associated with 222 significantly enriched or depleted GO BP terms after exclusion of dubious ORFs, including terms related to rRNA processing (GO:0006,364), ribosome assembly (GO:0042,255), and protein refolding (GO:0042,026), as well as depletion of broader metabolic regulatory categories such as regulation of metabolic process (GO:0019,222) (**Table S12**). By contrast, the heat shock condition displayed only 11 negatively enriched GO BP terms, mainly associated with broad metabolic-process categories (e.g. metabolic process, GO:0008,152; primary metabolic process, GO:0044,238; biosynthetic process, GO:0009,058) (**Table S13**). Comparison of these functional enrichments with those associated with the WGCNA modules showed that only seven GO BP terms were shared among heat ramp, heat shock, and the recurrent WGCNA signature (**Fig. 4C; Table S14**). Heat ramp retained 175 distinct GO BP terms, whereas heat shock did not display any specific GO signature under the selected criteria. Moreover, heat ramp and heat shock shared, respectively, 28 and 4 GO BP terms with the WGCNA-derived non-specific signature. Taken together, these results indicate that the heat-ramp response is characterized not only by a larger number of DEGs, but also by a much stronger condition-specific functional organization than heat shock.

Promoter motif enrichment analysis led to the same conclusion. In the heat-ramp condition, several TF-associated motifs were enriched, including motifs associated with Stb3, Dat1, Sfp1, Sum1, Azf1, Orc1, and Rgt1 (Fig. 4**D**). Some motif cores, particularly those associated with Sum1 and Stb3, were also recovered in the WGCNA-based analysis, indicating partial continuity between recurrent and condition-specific regulatory signatures. This overlap likely reflects redundancy and variant representation within the motif library, where closely related motifs can be reported as distinct entries with different enrichment statistics. However, heat-ramp-enriched hits also included additional motifs, notably those associated with Azf1, Orc1, Rgt1, and Dat1, that were not among the top WGCNA motif hits. This indicates that the heat-ramp response includes a regulatory component that extends beyond the recurrent non-specific signature.

This interpretation was further supported by the transcription-factor analysis. TF-associated regulons were identified independently from the 970 genes assigned to the WGCNA modules and from the DEG lists associated with heat ramp and heat shock using the YEASTRACT+ “Rank by TF” query. This tool ranks TFs according to the enrichment of their documented regulons in each input gene set relative to the whole yeast genome, and only associations with an FDR ≤ 0.01 were retained (**Table S15**). Many TFs were identified in the WGCNA and heat-ramp analyses, but their distribution was highly unbalanced. A total of 137 TFs were shared between the WGCNA modules and the heat-ramp condition (129 in the WGCNA∩HR overlap plus 8 shared across all three datasets), whereas heat shock yielded only 9 TFs in total. The relatively high number of TFs shared between WGCNA and heat ramp should be considered in light of the large input gene sets, comprising 970 WGCNA genes and 1633 heat-ramp DEGs, as well as the extensive overlap among documented TF target repertoires. These 137 TFs therefore represent candidate regulatory networks whose documented targets were significantly enriched in both gene sets, rather than TFs demonstrated to be active under the heat-ramp condition. Notably, 8 of these 9 HS TFs were located in the three-way intersection HR∩HS∩WGCNA, and only one TF was shared between heat ramp and heat shock outside WGCNA. Thus, convergence was strong between the recurrent WGCNA modules and the heat-ramp regulatory landscape, whereas the overlap between heat ramp and heat shock was numerically very limited relative to the HR TF repertoire (9 out of 148 TFs). This pattern is consistent with a much more restricted regulatory signature under the severe heat-shock endpoint and supports the view that heating kinetics influence transcriptional regulation, leading to partially divergent regulatory strategies under the two thermal regimes.

Overall, these results identify a much stronger condition-specific transcriptomic signature for heat ramp than for heat shock. While heat shock shares a limited core of responses with heat ramp and with the recurrent WGCNA-derived non-specific signature, the heat-ramp condition is distinguished by a much broader set of specific DEGs, a richer functional enrichment profile, and a more extensive regulatory signature. In the context of the bulk population-level framework adopted in this study, these findings indicate that gradual heating is associated with a more structured and distinctive transcriptional organization than abrupt heat shock at the endpoint analysed here.

### 3.4 DEGs highlighting discrepancies between phylostratigraphy and stress response gene clusters

**Fig. 5:**
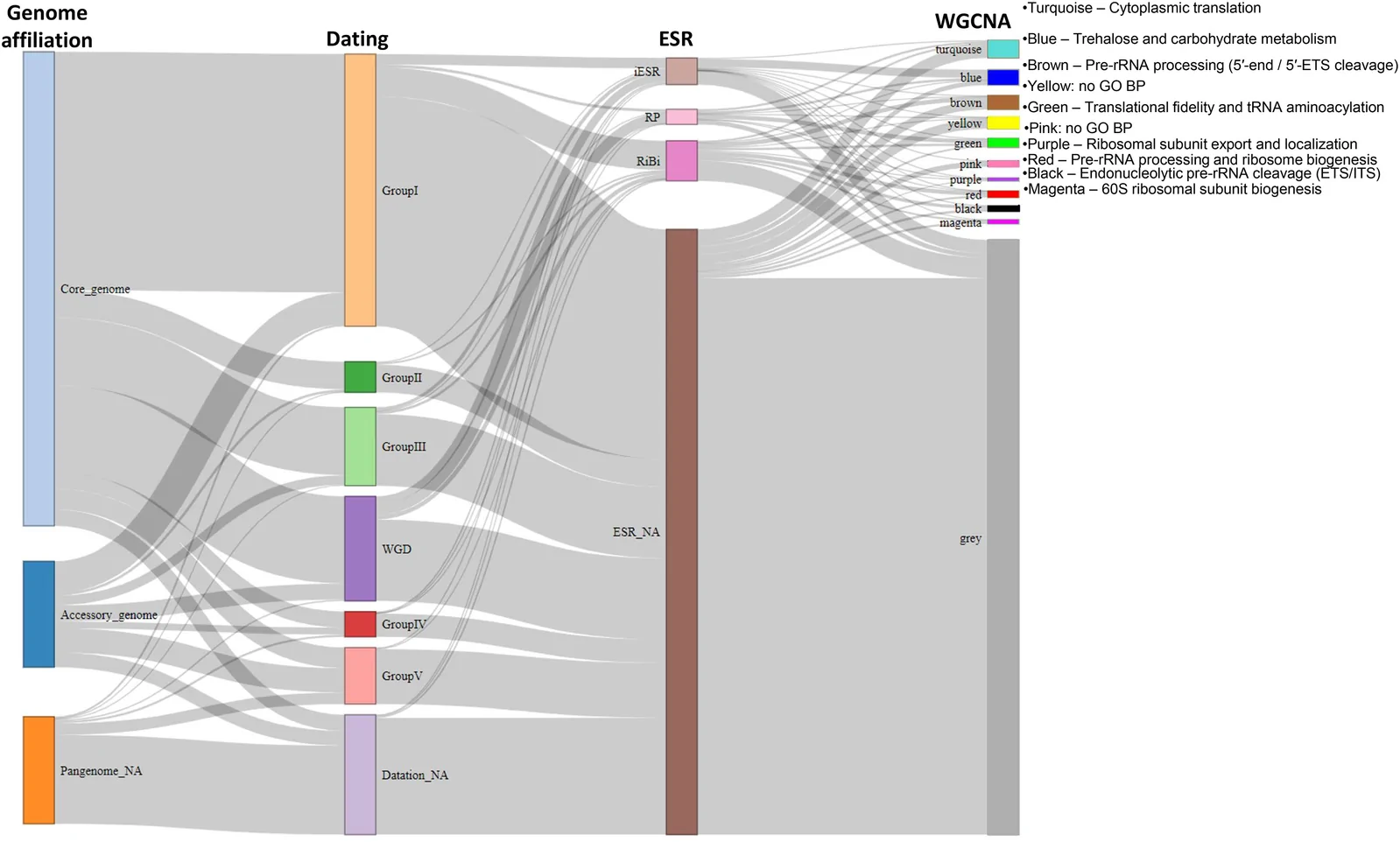
Sankey diagram showing the distribution of *S. cerevisiae* genes by genome affiliation, datation, ESR and WGCNA. **Genome affiliation**: clusters of core genome, accessory genome and unassigned genes (Pangenome_NA) based on Caudal et al. (2024). **Datation**: gene groups based on the phylostratigraphy approach developed by Doughty et al. (2020) Datation_NA refers to unassigned genes. **ESR**: clusters developed by Gasch et al. (2017). ESR_NA referred to genes not included in any ESR cluster (iESR, RiBi and RP). **WGCNA**: color modules based on the 15 yeast transcriptomes exposed to different heat treatments (present study, see Table 1). Grey module didn't correspond to any color module. The functional generic names (based on GO BP) of each WGCNA module color are mentionned close to the color name.

To place the ramp–shock divergence into a broader biological context, we compared our DEG signatures with two previously described frameworks: (i) the ESR/iESR stress-response clusters and (ii) gene-age groups defined by phylostratigraphy. The first framework corresponds to the Environmental Stress Response (ESR), including the induced ESR (iESR) cluster described by Gasch et al. (2017), as well as two major categories of genes generally repressed during stress: genes involved in ribosome biogenesis (RiBi) and genes encoding ribosomal proteins (RP), whereas the second is based on gene-age strata, including the evolutionarily young genes that emerged after the whole-genome duplication event and were highlighted by Doughty et al. (2020). In this classification, Groups I–III comprise progressively less widely conserved genes, WGD denotes genes retained after whole-genome duplication, and Groups IV and V correspond to *Saccharomyces* genus-specific and *S. cerevisiae*-specific genes, respectively. We also examined how these frameworks overlapped with the WGCNA modules identified in the present study. This comparison showed that these gene-grouping strategies only partially overlapped with one another (Fig. 5). The grey WGCNA category, corresponding to genes not assigned to a colored co-expression module, contained 51.3% of all iESR genes, 50.4% of all RiBi genes, and 28.9% of all RP genes. Among the colored modules, iESR genes were predominantly associated with the blue module, which contained 30.5% of the complete iESR set. RP genes were mainly represented in the green module (27.7%), although this proportion was very similar to that observed in the grey category. In contrast, RiBi genes assigned to colored modules were distributed across several modules, particularly brown (10.9%), red (10.2%), black (7.8%), and green (5.7%), consistent with the partitioning of ribosome-biogenesis and pre-rRNA-processing functions among several co-expression modules. In particular, many iESR genes were associated with older categories, Groups I-III and WGD, and with the core genome, whereas young genes (Groups IV and V) were often not assigned to classical stress-response clusters and frequently remained outside the recurrent WGCNA modules, notably within the grey module. These observations indicate that ESR-based, age-based, and co-expression-based classifications capture different dimensions of the yeast heat-stress response rather than a single unified structure.

We then examined the 15 transcriptomes through the pangenome, phylostratigraphy, and ESR frameworks. Across these datasets, no universal enrichment pattern linked thermotolerance or heat sensitivity to a single predefined gene category (**Fig. S5**). In particular, young genes (Groups IV and V) were not systematically associated with heat resistance, whereas iESR genes were more clearly connected to the heat-ramp response. Overall, these results indicate that the ramp–shock divergence is not systematically explained by preferential recruitment of evolutionarily young genes, but is more consistent with differential mobilization of conserved stress-response programs. Detailed distributions across pangenome, phylostratigraphic, and ESR categories are provided in the supplementary data (**Table S16**).

### 3.5 High involvement of chromosomal non-coding RNAs and limited contribution of mitochondrial coding mRNAs in heat ramp-induced thermal resistance

**Fig. 6:**
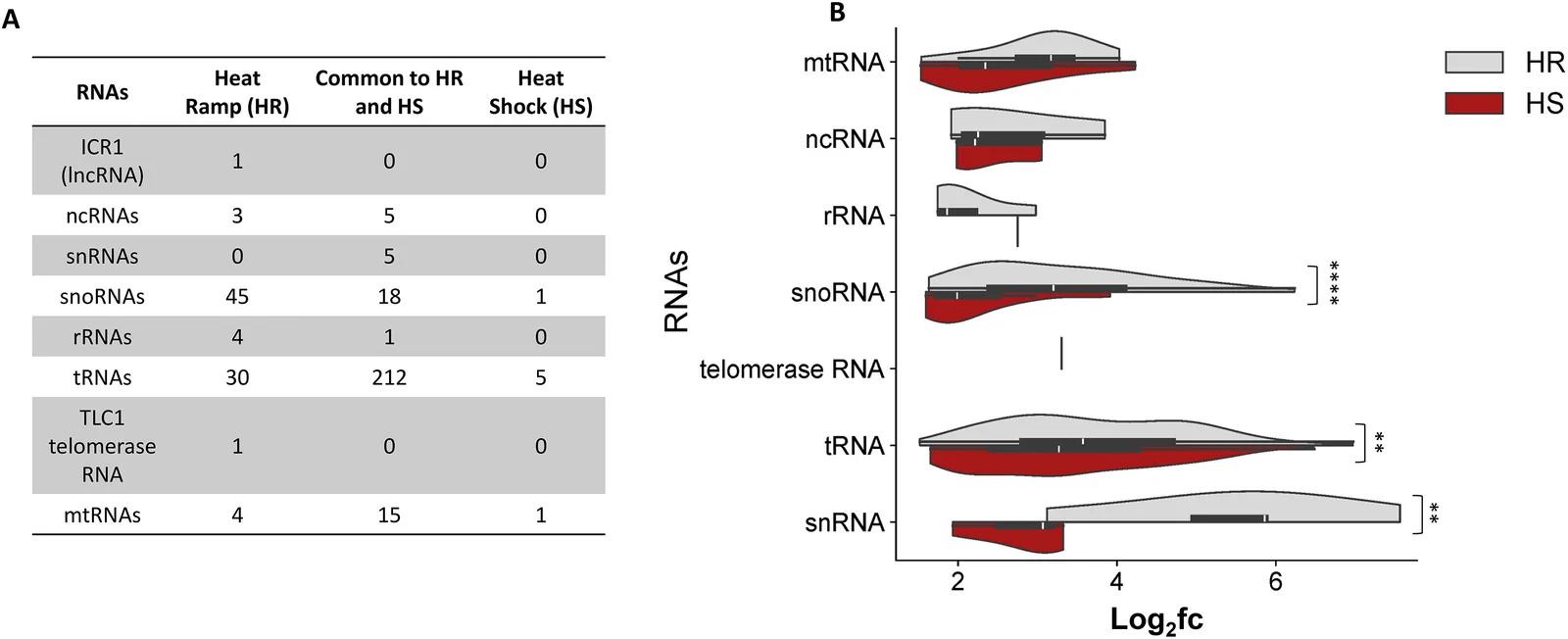
Non-coding and mitochondrial RNA signatures under heat ramp and heat shock **A:** Summary of differentially expressed RNAs (DEGs) detected in the RNA-seq dataset for each RNA biotype under heat ramp (HR) and heat shock (HS), and their overlap (HR-specific, HS-specific, and shared). RNA classes include: ncRNA (non-coding RNAs including RUF: RNA of Unknown Function), snRNAs (small nuclear RNAs), snoRNAs (small nucleolar RNAs), tRNAs (transfer RNAs), rRNAs (ribosomal RNAs), and mtRNAs (mitochondrial coding RNAs). Differential expression was assessed using the same thresholds as for poly(A)+ chromosomal coding mRNAs (see Methods). The identity, log_2_FC and adjusted p-values for each RNA are provided in Table S16. **B:** Half-violin/boxplot comparison of log2FC distributions between HR (grey) and HS (red) for each RNA biotype. Boxplots summarize median and interquartile range; violins depict the density of log2FC values within each class. Statistical significance of HR vs HS differences was assessed using a two-sided Student’s *t*-test when ANOVA residuals were normally distributed and variances were homogeneous; otherwise a two-sided Wilcoxon rank-sum test was applied (see Methods). Significance code: ns (p ≥ 0.05), * (p < 0.05), ** (p < 0.01), *** (p < 0.001), **** (p < 0.0001).

To further characterize the transcriptomic profiles associated with heat ramp and heat shock, we also examined RNA classes beyond chromosomal poly(A)+ coding mRNAs. In total, 394 chromosomal non-coding RNAs (ncRNAs) and 26 mitochondrial coding RNAs (mtRNAs) were analysed as part of the RNA-seq dataset. Differential expression was assessed using the same criteria as for chromosomal coding mRNAs (Fig. 6**A; Table S17**).

Across these RNA biotypes, 351 features were identified as differentially expressed, including 88 heat-ramp-specific, 256 shared between heat ramp and heat shock, and only 7 heat-shock-specific RNAs (Fig. 6**A**). Overall, DEGs were dominated by chromosomal non-coding RNAs (331/394), mainly tRNAs (n = 247) and snoRNAs (n = 64), whereas mtRNAs contributed a more limited fraction (20/26). HS-specific DEGs were restricted to one mtRNA (*Q0160*), one snoRNA (*snR59*), and five tRNAs (**Fig. 6A; Table S17**).

To compare the two heating regimes more directly, we analysed log_2_FC distributions for each RNA biotype (Fig. 6**B**). No significant difference was detected between heat ramp and heat shock for mtRNAs or for the global ncRNA class (Student’s *t*-test: mtRNA, p = 0.0603; ncRNA, p = 0.699). By contrast, significant differences were observed for snoRNAs (Wilcoxon, p = 4.14 × 10⁻⁵), tRNAs (Wilcoxon, p = 0.00332), and snRNAs (Student’s *t*-test, p = 0.00826) (Fig. 6**B**). These results indicate that, beyond chromosomal coding genes, heating kinetics also influence additional RNA classes, particularly those associated with RNA processing and translation-related functions.

Overall, these analyses support the same general conclusion as that obtained for chromosomal coding genes: heat ramp and heat shock differ not only in the amplitude of their transcriptional response, but also in the organization of specific RNA biotypes. However, compared with the coding-gene analyses, these RNA-class results should be viewed as a complementary layer of interpretation rather than as the primary basis for distinguishing the two thermal regimes.

## 4 Discussion

Bulk RNA-seq cannot resolve cell-to-cell heterogeneity, but it remains well suited to identifying reproducible population-level transcriptional patterns(Brettner et al., 2024; Gasch et al., 2017). Accordingly, our findings are interpreted as integrated outputs of stressed populations rather than as uniform single-cell states. This distinction is especially important for the severe heat-shock endpoint, for which the measured RNA signal may combine contributions from cultivable, injured, and non-cultivable cells.

Within this framework, heating kinetics emerged as a major determinant of transcriptomic organization. Gradual heating was associated with a broader and more functionally structured response than abrupt heat shock. The convergence of DEG, GO, promoter-motif, and TF-regulon enrichment analyses indicates that the two treatments differed not only in response magnitude but also in the organization of the population-level transcriptional program, with heat ramp showing stronger coordination of ribosome biogenesis, RNA processing, and proteostasis-related functions.

It is also important to emphasize that the heat-shock condition analysed here does not correspond to the mild or sublethal heat shocks commonly used to investigate transcriptional adaptation in yeast, which frequently involve shifts to 37 °C or 39 °C. Exposures to 50–51 °C nevertheless have a long-standing place in studies of severe heat stress, Hsp104-dependent thermotolerance, and genome-wide survival phenotypes in *S. cerevisiae* (Gibney et al., 2013; Lindquist and Kim, 1996; Sanchez et al., 1992; Teng et al., 2011). This temperature is also relevant to food-processing contexts, as recent challenge tests have examined the survival of *S. cerevisiae* in cocoa mass exposed to temperatures ranging from 50 to 65 °C (Maheneck et al., 2025). In the present study, both treatments reached the same final temperature of 50 °C and were followed by the same 30-min holding period, allowing the influence of heating kinetics to be assessed independently of the thermal endpoint. In the present study, the heat shock consisted of a rapid shift from 25 °C to 50 °C followed by 30 min at 50 °C, and was associated with a 70% reduction in cultivability, whereas the heat ramp caused no more than a 14% reduction under otherwise identical experimental conditions (Ragon et al., 2023). Accordingly, the corresponding heat-shock transcriptome should be interpreted as the population-level output of a severe endpoint condition rather than as the canonical response to a mild heat shock. This distinction is essential for interpreting the comparatively restricted heat-shock signature observed here and for understanding why the mostly milder public datasets included in the meta-analysis were used to identify recurrent heat-stress-associated patterns rather than as severity-matched comparators of the in-house experiments.

Cross-study WGCNA provided a complementary reference for interpreting this divergence. Despite differences among strains, platforms, and thermal protocols, recurrent modules were recovered around translation, ribosome biogenesis, RNA processing, and carbohydrate metabolism. These modules are best viewed as robust population-level patterns rather than identical cellular states across studies. The heat-ramp signature therefore appears to extend, rather than replace, this recurrent structure through additional genes, functions, and regulatory signals.

Several candidate regulators identified in the present analysis have previously been implicated in heat-shock survival in *S. cerevisiae*. Hsf1, Hac1, Swi6, Yap5, Msn1, Gcr2, and the SAGA component Spt3 were recovered in at least one of the WGCNA, heat-ramp, or heat-shock TF analyses. Jarolim et al. (2013) reported that reduced Hsf1 dosage and loss of Hac1, Swi6, or Spt3 impaired heat-shock survival, whereas Yap5, Msn1, and Gcr2 were linked to regulatory functions contributing to heat resistance. Hac1- and Yap5-associated regulons were identified across WGCNA, heat ramp, and heat shock, suggesting that unfolded-protein and iron/redox-homeostasis responses may contribute to a shared regulatory component across thermal regimes. By contrast, Hsf1-, Msn2-, and Msn4-associated regulons were recovered in WGCNA and heat ramp but not among the restricted set of heat-shock-associated TFs. Notably, Msn2 and Msn4 were not recovered in the deletion screen of Jarolim et al. (2013), which the authors attributed to their functional redundancy. These convergences support the biological relevance of the inferred regulatory landscape, although YEASTRACT+ enrichment identifies candidate regulons and does not demonstrate TF activation or a direct contribution to survival.

Within this broader regulatory landscape, the recovery of Sfp1-associated motifs in both the recurrent WGCNA structure and the heat-ramp-specific analysis is particularly noteworthy. In *S. cerevisiae*, Sfp1 links nutrient- and stress-responsive signalling to the transcriptional control of ribosomal-protein (RP) and ribosome-biogenesis (RiBi) genes (Jorgensen et al., 2004; Marion et al., 2004), providing a plausible connection with the prominent ribosome-biogenesis and RNA-processing signatures observed here. Functional diversification of this regulator is also evident in *Candida albicans*, where Sfp1 has been implicated in oxidative-stress regulation and in the repression of adhesion- and biofilm-associated genes (Chen and Lan, 2015; Lee et al., 2019). More directly, an SO₂-resistant evolved *S. cerevisiae* strain that acquired cross-resistance to oxidative stress and heat shock carried, among several genomic changes, a missense mutation in *SFP1* and displayed coordinated downregulation of ribosome-biogenesis and RNA-processing genes together with induction of stress-response, trehalose-biosynthesis, and carbohydrate-metabolism genes (Kısakesen et al., 2025). These observations support a possible overlap between thermal- and oxidative-stress adaptation and identify Sfp1 as a candidate regulator of the balance between growth-related and protective programmes. However, motif enrichment alone does not demonstrate Sfp1 binding or activation, and the available comparative evidence does not establish a causal role for Sfp1 under our experimental conditions.

Comparison with the ESR/iESR and phylostratigraphic frameworks showed that these classifications capture different dimensions of heat-stress biology(Doughty et al., 2020; Gasch et al., 2017). Their partial overlap with WGCNA, together with the association of many iESR genes with older and core-genome categories and the frequent exclusion of young genes from recurrent modules, argues against preferential recruitment of evolutionarily young genes as a general explanation. The ramp–shock divergence is instead more consistent with differential mobilization of conserved stress-response programs.

Analysis of non-coding and mitochondrial RNAs provided a complementary layer of evidence. Heating kinetics affected the log_2_FC distributions of snoRNAs, tRNAs, and snRNAs, consistent with broader effects on RNA processing and translation-related functions (Čáp and Palková, 2024; Parker et al., 2018). These RNA-class patterns support, but do not independently define, the distinction between heat ramp and heat shock. The five major spliceosomal snRNAs shared between the two treatments, U1 (snR19), U2 (LSR1), U4 (snR14), U5 (snR7-L), and U6 (snR6), were upregulated under both conditions but consistently showed higher log_2_FC values under heat ramp. Acute heat shock has previously been reported to moderately reduce the total abundance of spliceosomal snRNAs in yeast, independently of its more pronounced effects on snRNP (small nuclear ribonucleoprotein particles) organization (Bracken and Bond, 1999). The observed differences may therefore reflect heating-kinetics-dependent effects on snRNA synthesis, processing, or stability, although the present data do not provide information on snRNP assembly or functional splicing activity.

The biological significance of the heat-ramp signature lies in the coordinated preservation or reorganization of core cellular functions, in addition to the induction of canonical stress-protective genes. Enrichment of ribosome-biogenesis, RNA-processing, and proteostasis-related processes is consistent with maintenance of translation-associated capacity and protein homeostasis during gradual heating. This interpretation agrees with previous links between thermal adaptation, membrane behaviour, trehalose metabolism, stress proteins, and ribonucleoprotein dynamics (Davey and Guyot, 2020; Gibney et al., 2013; Guyot et al., 2015; Lindquist and Kim, 1996; Mihalik and Csermely, 2011; Mühlhofer et al., 2019; Ribeiro et al., 1997; Wallace et al., 2015).

These findings also illustrate the limits of transcriptomic inference. As reported by Gibney et al. (2013), heat-responsive genes overlapped only weakly with genes whose deletion impaired survival; our comparison with KO cultivability showed a similarly limited correspondence (**Fig. S6, Supp. Materials and Methods, Table S18**). Housekeeping genes were likewise not uniformly stable, particularly under heat ramp (**Table S19**). Transcript abundance should therefore not be treated as a direct proxy for causal contribution to thermotolerance, underscoring the need to combine transcriptomic, physiological, and functional-genetic approaches.

Our complementary physiological observations also reinforce this integrative interpretation. To further investigate the role of oxygen in the acquisition of thermotolerance, the wild-type strain BY4742 was exposed to conventional heat-stress protocols under both aerobic and anaerobic conditions (**Fig. S7A, Supp. Materials and Methods**). During the heat ramp, thermotolerance was observed irrespective of the presence of oxygen, indicating that oxygen is neither required for, nor a barrier to, its induction. In contrast, after heat shock, cells exhibited higher cultivability under anaerobic conditions. This result, consistent with Davidson and Schiestl (2001a, 2001b), suggests that oxygen contributes to oxidative stress, which in turn plays a role in heat-shock-induced cell death. It is also noteworthy that, under both heat ramp and heat shock conditions, ribonucleoprotein structures have been observed in association with the recruitment of the Pab1p protein (Ragon et al., 2023), although RNA-seq analysis did not reveal overexpression of the *PAB1* gene. Supporting this point, complementary experiments further showed that thermal stress kinetics had no measurable impact on the yeast’s capacity to consume dissolved oxygen after return to 25 °C, nor on mitochondrial membrane integrity (**Figs. S7B-C, Supp. Materials and Methods**). Together, these observations do not redefine the central transcriptomic conclusions, but they help constrain their biological interpretation and further highlight the value of integrating cell-physiology measurements with bulk transcriptomic analysis.

**Fig. 7:**
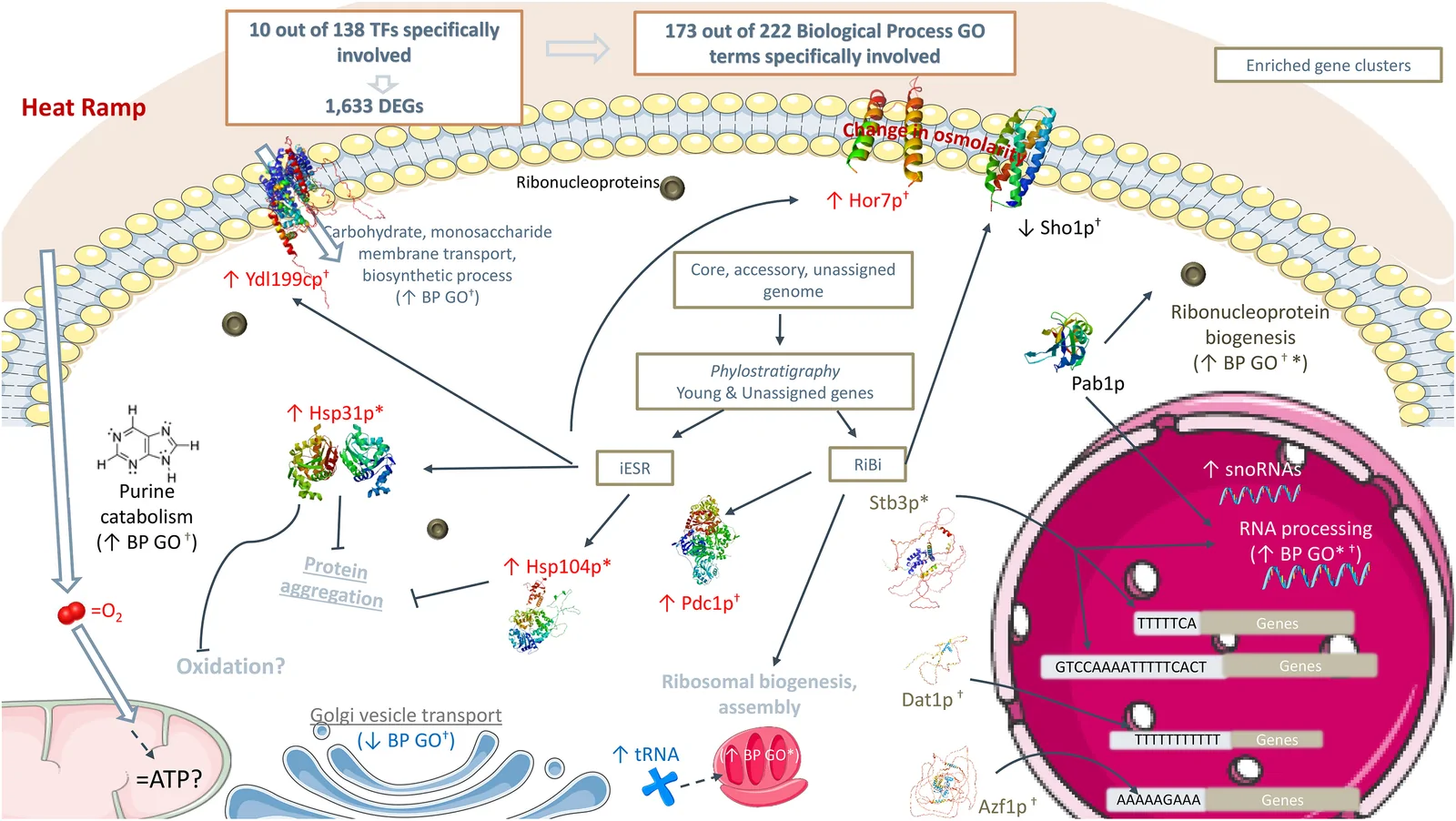
**Overview of the main resistance mechanisms activated during the Heat Ramp in *Saccharomyces cerevisiae* BY4742.** This conceptual summary represents the key cellular mechanisms involved in thermal resistance during the Heat Ramp in *S. cerevisiae* BY4742. It integrates both condition-specific (only Heat Ramp) and non-specific (WGCNA) gene clusters identified in the present study, as well as those previously described in the literature. Major associated Gene Ontology (GO) terms (excuding dubious ORFs), enriched promoter motifs, and transcription factors are also represented. In addition to transcriptomic data, relevant physiological traits are illustrated. This figure is not exhaustive but highlights the principal regulatory and functional responses contributing to cell survival under progressive heat stress including (^†^) specific, (*) non-specific and (^††^) shared (Heat Ramp and Heat Shock) signatures. Some images were provided by Servier Medical Art (https://smart.servier.com), licensed under CC BY 4.0 (https://creativecommons.org/licenses/by/4.0/).

**Fig. 8:**
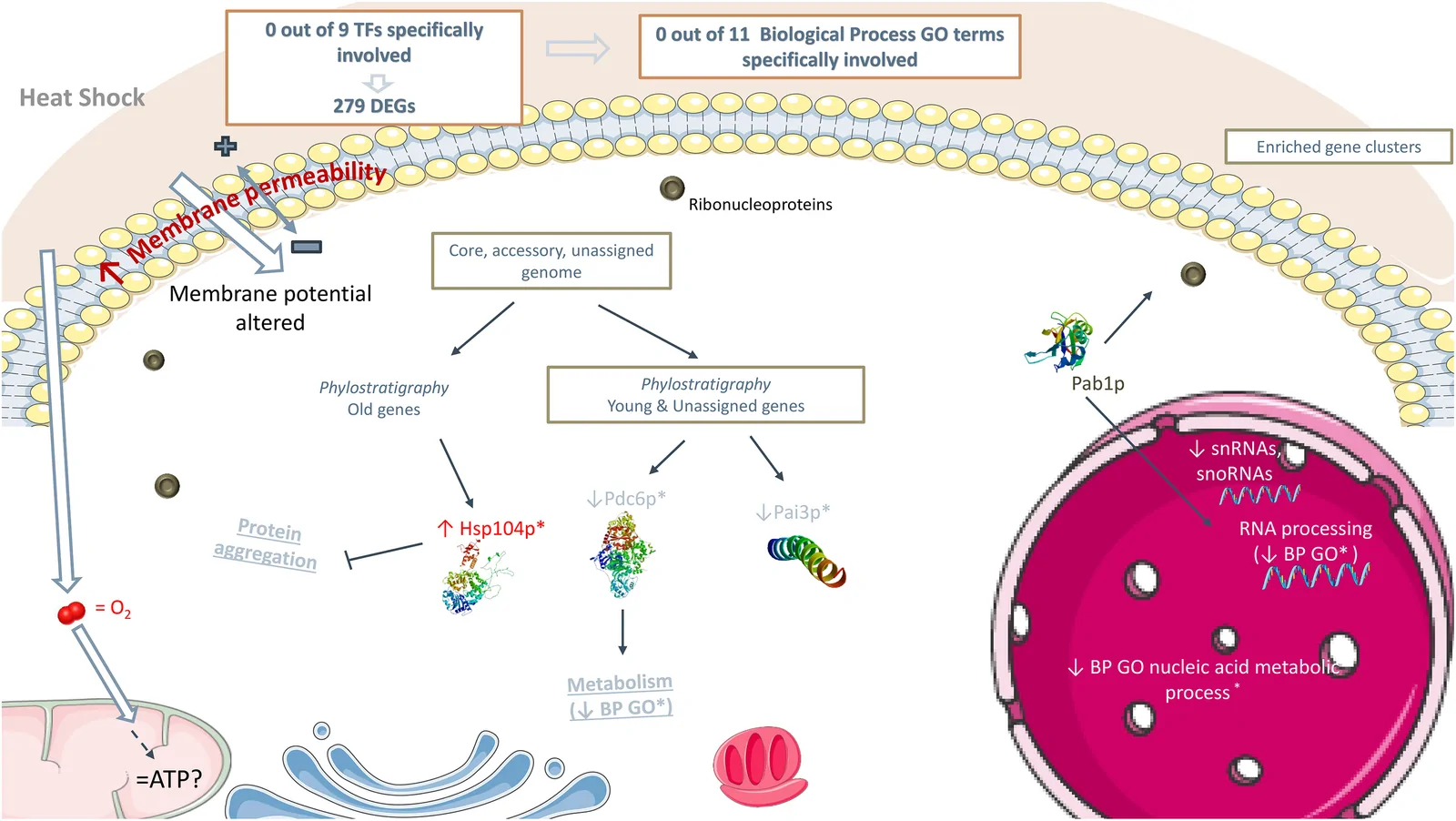
**Overview of the main resistance mechanisms activated during the Heat Shock in *Saccharomyces cerevisiae* BY4742.** This conceptual summary represents the key cellular mechanisms involved in thermal resistance during the Heat Ramp in *S. cerevisiae* BY4742. It integrates both condition-specific (only Heat Shock) and non-specific (WGCNA) gene clusters identified in the present study, as well as those previously described in the literature. Major associated Gene Ontology (GO) terms (excluding dubious ORFs) are also represented and interconnected. In addition to transcriptomic data, relevant physiological traits are illustrated. This figure is not exhaustive but highlights the principal regulatory and functional responses contributing to cell survival under a heat shock including (*) non-specific and (^††^) shared (Heat Ramp and Heat Shock) signatures. Some images were provided by Servier Medical Art (https://smart.servier.com), licensed under CC BY 4.0 (https://creativecommons.org/licenses/by/4.0/).

Taken together, the molecular and physiological results support the integrated view summarized in Fig. 7, Fig. 8. In the heat-ramp condition (Fig. 7), thermotolerance is associated with a broader and more structured transcriptional organization involving recurrent and condition-specific signatures linked to ribosome biogenesis, RNA processing, proteostasis, and membrane-associated functions, including the likely contribution of proteins such as Ydl199cp, Hor7p, and Sho1p. This interpretation is consistent with the idea that gradual heating better preserves cellular activity by limiting protein aggregation and supporting the maintenance or replenishment of functional proteins, in line with previous observations on heat-induced proteostasis in yeast (Mühlhofer et al., 2019). By contrast, the severe heat-shock endpoint (Fig. 8) is characterized by a much more restricted transcriptional signature, with weaker mobilization of clusters such as iESR and RiBi. In this context, the higher mortality observed after heat shock is consistent with a reduced capacity to activate protective mechanisms, including those involved in maintaining membrane integrity, which may contribute to increased plasma membrane permeability as previously reported (Martínez de Marañón et al., 1999). Thus, Fig. 7, Fig. 8 do not introduce additional results, but provide a compact synthesis of the distinct biological organizations associated with gradual and abrupt heating.

Several limitations should nevertheless be kept in mind. First, the study is based on bulk transcriptomic datasets and therefore cannot resolve the distribution of molecular states at the single-cell level. Second, the use of stationary-phase cultures also defines the physiological scope of the present study. Stationary-phase cells already exhibit adaptations associated with nutrient limitation, reduced proliferation, physiological heterogeneity, and enhanced resistance to environmental stress. Consequently, the transcriptional responses described here represent the effects of heating kinetics superimposed on a pre-adapted population. These responses may differ in amplitude and organization from those obtained with exponentially growing cultures, which generally display greater transcriptional dynamics but also greater thermal sensitivity. The present conclusions should therefore not be directly extrapolated to logarithmic-phase cells. Third, most public transcriptomes included in the meta-analysis were generated under milder heat-stress conditions than the severe 50 °C endpoint used in the in-house experiments. The resulting modules should therefore be interpreted as recurrent heat-stress-associated patterns across heterogeneous thermal contexts, rather than as severity-matched transcriptomic signatures at 50 °C. Additional transcriptomes obtained under comparable severe conditions will be required to assess the broader generalizability of these patterns. Fourth, the cross-study meta-analysis necessarily integrates heterogeneous designs, strains, platforms, and endpoint definitions. In this context, the WGCNA modules should be understood as recurrent patterns robust to heterogeneity, not as universal and exhaustive descriptors of every heat-stress response. Fifth, the severe heat-shock endpoint analysed here likely compresses multiple biological processes into a single integrated population-level signal, which may partly explain the limited condition-specific signature detected under that regime. These limitations do not invalidate the conclusions, but they define their scope.

## 5 Conclusion

Gradual heat ramp and abrupt heat shock do not simply represent two intensities of the same stress response in *Saccharomyces cerevisiae*. Rather, they are associated with distinct population-level transcriptomic organizations. By combining two controlled in-house datasets with thirteen heterogeneous public transcriptomes, this study identified recurrent non-specific heat-stress-associated modules as well as a much stronger condition-specific signature linked to gradual heating. Heat ramp was associated with broader differential expression, richer functional enrichments, and a more extensive regulatory signature than the severe heat-shock endpoint.

Importantly, the conclusions drawn here are framed at the level of bulk stressed populations. The heat-shock transcriptome analysed in this study corresponds to a severe endpoint condition associated with major loss of cultivability and should therefore be interpreted as an integrated output from physiologically heterogeneous subpopulations, rather than as the response of a uniform surviving fraction. Within this framework, the divergence between heat ramp and heat shock was more consistent with differential mobilization of conserved stress-response programs than with preferential recruitment of evolutionarily young genes.

More broadly, this work provides a transferable strategy for disentangling recurrent non-specific and condition-specific transcriptomic patterns in heterogeneous stressed populations. Beyond yeast, such an approach may prove useful for comparing bulk transcriptomic responses across diverse organisms, stress modalities, and experimental contexts, while preserving a clear distinction between shared stress-associated structure and condition-specific adaptation.

## Funding sources

Work carried out at UMR PAM (Dijon, France) was supported by the Regional Council of Bourgogne-Franche Comté, the “Fonds Européen de DEveloppement Régional (FEDER)” and AgroSup Dijon.

Lucie Bertheau and Jennifer Dumont were funded by the Regional Council of Bourgogne-Franche Comté and the “Fonds Européen de DEveloppement Régional (FEDER)” (PARI I – AGRALE 2 and PARI II - ALIM+ - 2015 programs)

## Supporting information

**Microsoft Excel file named** “Supplementary Processed Data”, containing the following spreadsheets:

Table S1. Processed data used to perform the weighted co-expression gene analysis (WGCNA) based on 15 transcriptomes sourced from various databases. Only Poly A+ mRNAs from chromosomal coding genes were considered.

Table S2. Genes ranked per WGCNA module colors based on 15 transcriptomes sources from various databases. For each gene, the ORF status (dubious or non-dubious) is indicated.

Table S3. Differentially expressed genes (DEGs) from Heat Ramp and Heat Shock RNA-seq. Transcriptomes were based on the expression of chromosomal coding genes. DEGs were defined using a stringent selection threshold of adjusted p-value (padj) ≤ 0.01 and absolute log_2_FC ≥ 1.5. For each gene, the ORF status (dubious or non-dubious) is indicated. This table also reports the proportion of dubious and non-dubious ORFs within each DEG set.

Table S4. Heat Ramp RNA-seq raw data. Data are openly available in Gene Expression Omnibus at GSE276309. The ORF status (dubious or non-dubious) is specified for each gene. Sensitivity analyses showing the number of differentially expressed genes (DEGs) as a function of the absolute log_2_FC (|log_2_FC|). The blue curves correspond to a stringent selection threshold (adjusted p-value, padj ≤ 0.01), whereas the orange curves correspond to a less stringent threshold (padj ≤ 0.05).

Table S5. Heat Shock RNA-seq raw data. Data are openly available in Gene Expression Omnibus at GSE276309. The ORF status (dubious or non-dubious) is specified for each gene. The sensitivity curves were produced as specified in the legend of Table S4.

Table S6. Differentially expressed genes (DEGs) from Heat Ramp and Heat Shock RNA-seq. Transcriptomes were based on the expression of chromosomal coding genes. DEGs were defined using a stringent selection threshold of adjusted p-value (padj) ≤ 0.05 and absolute log_2_FC ≥ 1. For each gene, the ORF status (dubious or non-dubious) is indicated. This table also reports the proportion of dubious and non-dubious ORFs within each DEG set.

Table S7. WGCNA Module-Trait Correlation Analysis. Module-trait relationships were analyzed using Spearman correlation with Benjamini-Hochberg FDR correction for multiple testing. Three phenotypes were considered: viability, origin and ploidy. The corresponding R script is provided.

Table S8. WGCNA Analysis of Gene Ontology (GO) Biological Processes (BP). Only GO BP Terms with FDR ≤ 0.01 were considered for subsequent analysis. Transcriptomes were based on the expression of chromosomal coding genes. Dubious ORFs were excluded from the DEGs list before running GO analysis. This table also reports GO Biological Process (BP) terms that were enriched in two or more WGCNA modules (dubious ORFs excluded). For each GO BP term, we indicate the module colors in which it was enriched and the corresponding enrichment statistics. -log_10_(FDR) values are provided per module (0 indicates that the GO term was not reported as enriched for that module in the selected list). This overlap summary complements Fig. 2B-C by providing the identity (qualitative information) of the shared GO BP terms across modules.

Table S9. Protein-Protein Interactions (PPIs) matrix. The matrix was generated using the STRING database and associated tools based on WGCNA (transcriptomes were based on the expression of chromosomal coding genes).

Table S10. Promoter motif enrichment performed separately for each WGCNA module (excluding dubious ORFs). This table compiles the full promoter motif enrichment outputs obtained independently for each module color using module-specific DEG lists. For each module, enriched motifs, predicted transcription factors (TFs), TF families, and associated significance metrics (P-value, FDR, score) are reported as returned by the enrichment tool. Cells highlighted in pink correspond to motifs with FDR between 1 × 10⁻⁵ and 1 × 10⁻²⁰ (threshold range retained for summarization in Fig. 3E). Repeated occurrences of closely related motifs may occur within a given module due to motif variants tested by the enrichment database; FDR values are adjusted within each module (Benjamini–Hochberg). ‘Score’ is the internal ranking metric reported by the motif-enrichment tool; all statistical significance decisions were based on FDR.

Table S11. Specific and common DEGs including dubious ORFs in *S. cerevisiae* BY4742 from the Heat Ramp, Heat Shock and WGCNA list.

Table S12. Heat Ramp Analysis of Gene Ontology (GO) Biological Processes (BP). Only GO BP Terms with FDR ≤ 0.01 were considered for subsequent analysis. Transcriptomes were based on the expression of chromosomal coding genes. Dubious ORFs were excluded from the DEGs list before running GO analysis. DEGs were defined using a stringent selection threshold of adjusted p-value (padj) ≤ 0.01 and absolute log_2_FC ≥ 1.5

Table S13. Heat Shock Analysis of Gene Ontology (GO) Biological Processes (BP). Only GO BP Terms with FDR ≤ 0.01 were considered for subsequent analysis. Transcriptomes were based on the expression of chromosomal coding genes. Dubious ORFs were excluded from the DEGs list before running GO analysis. DEGs were defined using a stringent selection threshold of adjusted p-value (padj) ≤ 0.01 and absolute log_2_FC ≥ 1.5

Table S14. Specific and common Gene Ontology (GO) Biological Processes (BP) terms in *S. cerevisiae* BY4742 from the Heat Ramp, Heat Shock and WGCNA list.

Table S15. Transcription factors (TFs) identified in WGCNA modules, DEGs from the Heat Ramp, and DEGs from the Heat Shock condition. TFs were identified using the “Rank by TF” query in YEASTRACT+, which groups an input gene list according to its documented regulators and ranks TFs according to the enrichment of their regulons in the input dataset relative to the whole yeast genome. The “Check for all TFs” option was used. Only TFs with an FDR ≤ 0.01 were considered. TFs were retrieved from YEASTRACT+ on 13 May 2025. Documented regulations were filtered by combined DNA-binding and expression evidence, and TF acting as activator or inhibitor. The *p.adjust* function in R with the Benjamini–Hochberg method was used to correct raw p-values. The resulting TFs correspond to significantly enriched documented regulons and should not be interpreted as TFs experimentally demonstrated to be active or differentially expressed under the corresponding condition.

Table S16. Gene-level classifications used to construct the Sankey diagram presented in Fig. 5. For each gene, the table reports its annotation, ESR-related category, WGCNA module assignment, orthology-based gene group, gene-age category, and pangenome assignment. ESR-related categories were obtained from Table S8 of Gasch et al. (2017) and comprise the induced Environmental Stress Response (iESR), ribosome-biogenesis (RiBi), and ribosomal-protein (RP) clusters. Orthology-based gene groups and gene-age categories, including Groups I-V and the whole-genome duplication (WGD) category, were obtained from Doughty et al. (2020). Pangenome and gene-origin assignments were obtained from Caudal et al. (2023). “NA” indicates that no corresponding assignment was available.

Table S17. DEGs from non-coding chromosomal and coding mitochondrial genes. See Tables S4 and S5 for RNA-seq raw data.

Table S18. Yeast deletion mutants strains (KO): characteristics and sources.

Table S19. Gene expression of housekeeping genes in *Saccharomyces cerevisiae* under heat ramp and heat shock conditions. Log₂ fold-change and padj values were reported from Tables S4 and S5 for the heat ramp and heat shock, respectively. Genes highlighted in yellow correspond to differentially expressed genes (DEGs).

Figure S1. Global transcriptomic analysis based on Principal Component Analysis (PCA) after a shock and a ramp from 25 °C to 50 °C in YPD medium. PCA based on differentially expressed chromosomal coding genes per sample was performed in R Studio environment (version 1.2.5033) using FactoMineR, factoextra and Factoshiny packages. (A) PCA with significant clustering, (B) dendograms, (C) Percentage of variance explained by each of the first ten principal components obtained from PCA of the quality-indicator value distributions (control maintained few seconds at 25 °C, shock, ramp).

Figure S2. Evaluation of batch effects prior to correction. (A) Principal Component Analysis (PCA) of all samples based on log2 fold change values, colored by sequencing method and laboratory of origin. (B) Distribution of PC1 and PC2 across sequencing methods and laboratories, shown as boxplots. (C) Summary table of statistical tests performed on PC1 and PC2: Shapiro-Wilk test for normality of residuals, followed by either ANOVA (with Levene’s test) or Kruskal-Wallis (with Fligner-Killeen test) depending on residual distribution. No significant batch effects were detected at α = 0.01.

Figure S3. Evaluation of batch effects after ComBat correction (mean.only = TRUE). (A) PCA plot after correction, colored by sequencing method and laboratory. (B) Boxplots of PC1 and PC2 across methods and laboratories. (C) Statistical summary of residuals and variance homogeneity tests, showing the disappearance of batch effects post-correction.

Figure S4. WGCNA scale independence and mean connectivity. Transcriptomes were based on the expression of chromosomal coding genes.

Figure S5. Circos visualisations of the transcriptomic features of 15 strains of *Saccharomyces cerevisiae*. Transcriptomes were viewed through the pangenome, phylostratigraphy and ESR by considering three distributions from the outer to the inner circle: fold enrichment (see Materials and Methods section for calculation details), DEGs counts (DEGs names) and total gene counts (total counts names). Gene groups or clusters across the whole genome are shown in the inner circle. The origin and thermal adaptation associated with the transcriptome are shown around the circle and their meaning has been specified in the Legend panel. Yeast strains marked with an asterisk (*) showed a certain degree of cell death under the heat treatment described in the corresponding paper (see Table 1). A: Transcriptomes viewed through the pangenome, considering core genome, accessory genome and unassigned gene groups. B: Transcriptomes viewed through the phylostratigraphy by considering gene age groups (I, II, III, WGD, IV and V). C: Transcriptomes viewed through ESR clusters by considering RP, RiBi and iESR clusters. The "Gene expression" panel shows the individual log_2_FC change values of all expressed genes; each dot corresponds to one gene, allowing visualization of the overall distribution and direction of expression changes.

Figure S6. Expression levels of selected genes in *S. cerevisiae* BY4742 wild-type strain as a function of the mean cultivability of corresponding gene deletion mutants. The pink area indicates genes that are not differentially expressed (−1.5 < log₂FC < 1.5). The brown line represents the mean cultivability measured after Heat Shock, and the grey line corresponds to the mean cultivability observed after the Heat Ramp (Ragon et al., 2023). The colony forming unit method (CFU) was used to enumerate yeasts. Immediately after the application of the heat stresses (or after washing samples with fresh YPD medium in the case of control samples), 10 µL of four replicates of the appropriate decimal dilution of the cell suspension were dropped on YPD-agar medium. CFU were counted after incubation for 48 h at 25 °C. At least four independent cultures of yeast were tested.

Figure S7. Impacts of energy metabolism on the thermoresistance response of BY4742 strain. (A) According to experimental procedures, the wild-type strain BY4742 was exposed to a heat slope or heat shock combined with presence or absence of oxygen. Cell enumeration corresponds to a percentage relative to the control of the number of colonies counted following treatments. Means ± standard errors of at least four independent replicates of cell enumeration measurements are presented in each case. The mean values of percentage of cell enumeration after heat treatments were compared by ANOVA followed by Scheffe test; different letters indicate significant differences (P < 0.05).

(B) Oxygen consumption of the wild-type strain BY4742 after heat slope or heat shock treatments. Following heat stresses application, instantaneous respiration rates were measured on YPD medium maintained at 25 °C during 15 min of recording, and cultivability tests were realized in parallel from the same samples. R corresponds to a ratio of unstressed control of the velocity of the disappearance of oxygen by UFC. Means ± standard errors of at three independent replicates of O_2_ measurements are presented. The mean values of each treatment were compared together by a *t*-test, NS for not significant.

(C) Appreciation of mitochondrial potential and integrity of the wild-type strain BY4742 after heat slope or heat shock treatments. Flow cytometric analysis were realized from control or stressed yeast cells diluted in PBS at 10^6^ cells.mL^-1^ and stained with Rhodamine 123 at 0.01 µM. Determined by live cells, M1 is the region where the fluorescence signal is negative. R value (FL1-A / FSC-A ratio) has been calculated to normalize the intensity of the fluorescent signal that can vary with the size of yeast as proposed by Ludovico et al. (2001). () unstressed yeasts, () yeasts exposed to the heat slope and then maintained 50 min at 50 °C, () yeasts exposed to a heat shock and then maintained 30 min at 50 °C, () boiled yeasts.

At least four independent measurements were performed for each of the conditions tested. Statistical analyses were performed by the statistical R software [version 3.2.4 Revised (2016–03-r70336)]. For each variable, the normality of the distribution was tested by a Shapiro-Wilk test. Results are expressed as means of percentage of the unstressed control of CFU counted after treatments, with their standard errors and compared by *t*-test or ANOVA followed by Scheffe test. Alpha risk was 0.05. All statistical tests were considered significant at P < 0.05.

## Declaration of competing interest

The authors declare that they have no known competing financial interests or personal relationships that could have appeared to influence the work reported in this paper.

## Data Availability

All relevant data are available from the authors on request. The processed data, collected from various databases, are provided in electronic format as a Microsoft Excel file named 'Supplementary Processed Data', containing the following spreadsheets: **Tables S1-S19**. The original RNA-seq data presented in the study are openly available in Gene Expression Omnibus at GSE276309.

## Appendix Supplementary materials

Image, application 1

Image, application 2

Image, application 3

Image, application 4

Image, application 5

Image, application 6

Image, application 7

Image, application 8

Image, application 9

## References

[bib0001] Bankapalli K., Saladi S., Awadia S.S., Goswami A.V., Samaddar M., D’Silva P. (2015). Robust glyoxalase activity of Hsp31, a ThiJ/DJ-1/PfpI family member protein, is critical for oxidative stress resistance in Saccharomyces cerevisiae. J. Biol. Chem..

[bib0002] Bardou P., Mariette J., Escudié F., Djemiel C., Klopp C. (2014). jvenn: an interactive Venn diagram viewer. BMC Bioinform..

[bib0003] Berry D.B., Gasch A.P. (2008). Stress-activated genomic expression changes serve a preparative role for impending stress in yeast. Mol. Biol. Cell.

[bib0004] Berry D.B., Guan Q., Hose J., Haroon S., Gebbia M., Heisler L.E., Nislow C., Giaever G., Gasch A.P. (2011). Multiple means to the same end: The Genetic basis of acquired stress resistance in yeast. PLoS Genet..

[bib0005] Bolger A.M., Lohse M., Usadel B. (2014). Trimmomatic: a flexible trimmer for Illumina sequence data. Bioinformatics.

[bib0006] Bracken A.P., Bond U. (1999). Reassembly and protection of small nuclear ribonucleoprotein particles by heat shock proteins in yeast cells. RNA.

[bib0007] Brettner L., Eder R., Schmidlin K., Geiler-Samerotte K. (2024). An ultra high-throughput, massively multiplexable, single-cell RNA-seq platform in yeasts. Yeast.

[bib0008] Čáp M., Palková Z. (2024). Non-coding RNAs: Regulators of stress, ageing, and developmental decisions in yeast?. Cells.

[bib0009] Caspeta L., Chen Y., Ghiaci P., Feizi A., Buskov S., Hallstrom B.M., Petranovic D., Nielsen J. (2014). Altered sterol composition renders yeast thermotolerant. Science (1979).

[bib0010] Caudal É., Loegler V., Dutreux F., Vakirlis N., Teyssonnière É., Caradec C., Friedrich A., Hou J., Schacherer J. (2024). Pan-transcriptome reveals a large accessory genome contribution to gene expression variation in yeast. Nat. Genet..

[bib0011] Chen H.-F., Lan C.-Y. (2015). Role of SFP1 in the regulation of Candida albicans biofilm formation. PLoS. One.

[bib0012] Davey H., Guyot S. (2020). Estimation of microbial viability using flow cytometry. Curr. Protoc. Cytom..

[bib0013] Davidson J.F., Schiestl R.H. (2001). Mitochondrial respiratory electron carriers are involved in oxidative stress during heat stress in Saccharomyces cerevisiae. Mol. Cell Biol..

[bib0014] Davidson J.F., Schiestl R.H. (2001). Cytotoxic and genotoxic consequences of heat stress are dependent on the presence of oxygen in Saccharomyces cerevisiae. J. Bacteriol..

[bib0015] Doughty T.W., Domenzain I., Millan-Oropeza A., Montini N., de Groot P.A., Pereira R., Nielsen J., Henry C., Daran J.-M.G., Siewers V., Morrissey J.P. (2020). Stress-induced expression is enriched for evolutionarily young genes in diverse budding yeasts. Nat. Commun..

[bib0016] Eng K.H., Kvitek D.J., Keleş S., Gasch A.P. (2010). Transient genotype-by-environment interactions following environmental shock provide a source of expression variation for essential genes. Genetics.

[bib0017] Engel S.R., Dietrich F.S., Fisk D.G., Binkley G., Balakrishnan R., Costanzo M.C., Dwight S.S., Hitz B.C., Karra K., Nash R.S., Weng S., Wong E.D., Lloyd P., Skrzypek M.S., Miyasato S.R., Simison M., Cherry J.M. (2014). The reference genome sequence of saccharomyces cerevisiae: then and now. G3 Genes|Genomes|Genetics.

[bib0018] Fligner M.A., Killeen T.J. (1976). Distribution-free two-sample tests for scale. J. Am. Stat. Assoc..

[bib0019] Franz M., Lopes C.T., Fong D., Kucera M., Cheung M., Siper M.C., Huck G., Dong Y., Sumer O., Bader G.D. (2023). Cytoscape.js 2023 update: a graph theory library for visualization and analysis. Bioinform.

[bib0020] Gao L., Liu Y., Sun H., Li C., Zhao Z., Liu G. (2016). Advances in mechanisms and modifications for rendering yeast thermotolerance. J. Biosci. Bioeng..

[bib0021] Gasch A.P., Spellman P.T., Kao C.M., Carmel-Harel O., Eisen M.B., Storz G., Botstein D., Brown P.O. (2000). Genomic expression programs in the response of yeast cells to environmental changes. Mol. Biol. Cell.

[bib0022] Gasch A.P., Yu F.B., Hose J., Escalante L.E., Place M., Bacher R., Kanbar J., Ciobanu D., Sandor L., Grigoriev I.V., Kendziorski C., Quake S.R., McClean M.N. (2017). Single-cell RNA sequencing reveals intrinsic and extrinsic regulatory heterogeneity in yeast responding to stress. PLoS Biol..

[bib0023] Ge S.X., Jung D., Yao R. (2020). ShinyGO: a graphical gene-set enrichment tool for animals and plants. Bioinformatics.

[bib0024] Ge S.X., Son E.W., Yao R. (2018). iDEP: an integrated web application for differential expression and pathway analysis of RNA-Seq data. BMC Bioinform..

[bib0025] Gervais P., Martínez de Marañón I. (1995). Effect of the kinetics of temperature variation on Saccharomyces cerevisiae viability and permeability. Biochim. Biophys. Acta.

[bib0026] Gibney P.A., Lu C., Caudy A.A., Hess D.C., Botstein D. (2013). Yeast metabolic and signaling genes are required for heat-shock survival and have little overlap with the heat-induced genes. Proc. Natl. Acad. Sci. U. S. A..

[bib0027] Goffeau A., Barrell B.G., Bussey H., Davis R.W., Dujon B., Feldmann H., Galibert F., Hoheisel J.D., Jacq C., Johnston M., Louis E.J., Mewes H.W., Murakami Y., Philippsen P., Tettelin H., Oliver S.G. (1996). Life with 6000 genes. Science (1979).

[bib0028] Guyot S., Ferret E., Gervais P. (2005). Responses of Saccharomyces cerevisiae to thermal stress. Biotechnol. Bioeng..

[bib0029] Guyot S., Gervais P., Young M., Winckler P., Dumont J., Davey H. (2015). Surviving the heat: Heterogeneity of response in Saccharomyces cerevisiae provides insight into thermal damage to the membrane. Environ. Microbiol..

[bib0030] Hill L., Guyot S., Bertheau L., Davey H. (2024). Calorie restriction decreases competitive fitness in Saccharomyces cerevisiae following heat stress. Microorganisms.

[bib0031] Huang D.W., Sherman B.T., Lempicki R.A. (2009). Systematic and integrative analysis of large gene lists using DAVID bioinformatics resources. Nat. Protoc..

[bib0032] Jarolim S., Ayer A., Pillay B., Gee A.C., Phrakaysone A., Perrone G.G., Breitenbach M., Dawes I.W. (2013). Saccharomyces cerevisiae genes involved in survival of heat shock. G3 (Bethesda).

[bib0033] Johnson W.E., Li C., Rabinovic A. (2007). Adjusting batch effects in microarray expression data using empirical Bayes methods. Biostat.

[bib0034] Jorgensen P., Rupeš I., Sharom J.R., Schneper L., Broach J.R., Tyers M. (2004). A dynamic transcriptional network communicates growth potential to ribosome synthesis and critical cell size. Genes Dev..

[bib0035] Jung J., Mok C., Lee W., Jang W. (2017). Meta-analysis of microarray and RNA-Seq gene expression datasets for carcinogenic risk: an assessment of Bisphenol. A. Mol. Cell. Toxicol..

[bib0036] Kısakesen H.İ., Canbay Z.B., Korkmaz A.K., Topaloğlu A., Esen Ö., Arslan M., Holyavkin C., Çakar Z.P. (2025). Evolutionary engineering and molecular characterization of a sulfur dioxide-stress-resistant <I>Saccharomyces cerevisiae</i>strain. Fermentation.

[bib0037] Langfelder P., Horvath S. (2008). WGCNA: an R package for weighted correlation network analysis. BMC. Bioinformatics.

[bib0038] Langfelder P., Mischel P.S., Horvath S. (2013). When is hub gene selection better than standard meta-analysis?. PLoS One.

[bib0039] Langmead B., Salzberg S.L. (2012). Fast gapped-read alignment with Bowtie 2. Nat. Methods.

[bib0040] Lê S., Josse J., Husson F. (2008). FactoMineR: An R package for multivariate analysis. J. Stat. Softw..

[bib0041] Lee S.-Y., Chen H.-F., Yeh Y.-C., Xue Y.-P., Lan C.-Y. (2019). The transcription factor Sfp1 regulates the oxidative stress response in Candida albicans. Microorganisms.

[bib0042] Leek J.T., Johnson W.E., Parker H.S., Jaffe A.E., Storey J.D. (2012). The sva package for removing batch effects and other unwanted variation in high-throughput experiments. Bioinform.

[bib0043] Lindquist S., Kim G. (1996). Heat-shock protein 104 expression is sufficient for thermotolerance in yeast. Proc. Natl. Acad. Sci. U. S. A..

[bib0044] López-Malo A., Guerrero S., Alzamora S.M. (1999). Saccharomyces cerevisiae thermal inactivation kinetics combined with ultrasound. J. Food Prot..

[bib0045] Love M.I., Huber W., Anders S. (2014). Moderated estimation of fold change and dispersion for RNA-seq data with DESeq2. Genome Biol..

[bib0046] Ludovico P., Sansonetty F., Corte-Real M. (2001). Assessment of mitochondrial membrane potential in yeast cell populations by flow cytometry. Microbiol.

[bib0047] Maheneck M.V., Nzouengo Tchounda R.J., Essia-Ngang J.J., Sado Kamdem S.L. (2025). Microbial quality during cocoa mass production and effect of its storage conditions on the survival of Klebsiella oxytoca, *Escherichia coli*, Saccharomyces cerevisiae and Aspergillus brasiliensis. J. Food Qual..

[bib0048] Marion R.M., Regev A., Segal E., Barash Y., Koller D., Friedman N., O’Shea E.K. (2004). Sfp1 is a stress- and nutrient-sensitive regulator of ribosomal protein gene expression. Proc. Natl. Acad. Sci. U. S. A..

[bib0049] Martínez de Marañón I., Chaudanson N., Joly N., Gervais P. (1999). Slow heat rate increases yeast thermotolerance by maintaining plasma membrane integrity. Biotechnol. Bioeng..

[bib0050] Mi H., Ebert D., Muruganujan A., Mills C., Albou L.-P., Mushayamaha T., Thomas P.D. (2021). PANTHER version 16: a revised family classification, tree-based classification tool, enhancer regions and extensive API. Nucleic. Acids. Res..

[bib0051] Mi H., Muruganujan A., Huang X., Ebert D., Mills C., Guo X., Thomas P.D. (2019). Protocol Update for large-scale genome and gene function analysis with the PANTHER classification system (v.14.0). Nat. Protoc..

[bib0052] Mi H., Muruganujan A., Thomas P.D. (2013). PANTHER in 2013: modeling the evolution of gene function, and other gene attributes, in the context of phylogenetic trees. Nucleic. Acids. Res..

[bib0053] Mi H., Thomas P. (2009). PANTHER Pathway: an ontology-based pathway database coupled with data analysis tools. Methods Mol. Biol..

[bib0054] Mihalik A., Csermely P. (2011). Heat shock partially dissociates the overlapping modules of the yeast protein-protein interaction network: a systems level model of adaptation. PLoS. Comput. Biol..

[bib0055] Mir S.S., Fiedler D., Cashikar A.G. (2009). Ssd1 is required for thermotolerance and Hsp104-mediated protein disaggregation in Saccharomyces cerevisiae. Mol. Cell Biol..

[bib0056] Mooney M., Bond J., Monks N., Eugster E., Cherba D., Berlinski P., Kamerling S., Marotti K., Simpson H., Rusk T., Tembe W., Legendre C., Benson H., Liang W., Webb C.P. (2013). Comparative RNA-Seq and microarray analysis of gene expression changes in B-cell lymphomas of Canis familiaris. PLoS One.

[bib0057] Morozov I.I., Petin V.G., Dubovick B.V. (1997). Influence of tonicity and chloramphenicol on hyperthermic cytotoxicity and cell permeability under various heating rates. Int. J. Hyperthermia.

[bib0058] Mühlhofer M., Berchtold E., Stratil C.G., Csaba G., Kunold E., Bach N.C., Sieber S.A., Haslbeck M., Zimmer R., Buchner J. (2019). The heat shock response in yeast maintains protein homeostasis by chaperoning and replenishing proteins. Cell Rep..

[bib0059] Nakamura T., Yamamoto M., Saito K., Ando A., Shima J. (2014). Identification of a gene, FMP21, whose expression levels are involved in thermotolerance in Saccharomyces cerevisiae. AMB Express.

[bib0060] Niemira M., Collin F., Szalkowska A., Bielska A., Chwialkowska K., Reszec J., Niklinski J., Kwasniewski M., Kretowski A. (2020). Molecular signature of subtypes of non-small-cell lung cancer by large-scale transcriptional profiling: identification of key modules and genes by weighted gene co-expression network analysis (WGCNA). Cancers. (Basel).

[bib0061] Parker S., Fraczek M.G., Wu J., Shamsah S., Manousaki A., Dungrattanalert K., Almeida R.A., de, Invernizzi E., Burgis T., Omara W., Griffiths-Jones S., Delneri D., O’Keefe R.T. (2018). Large-scale profiling of noncoding RNA function in yeast. PLoS Genet..

[bib0062] Ragon M., Bertheau L., Dumont J., Bellanger T., Grosselin M., Basu M., Pourcelot E., Horrigue W., Denimal E., Marin A., Vaucher B., Berland A., Lepoivre C., Dupont S., Beney L., Davey H., Guyot S. (2023). The Yin-Yang of the green fluorescent protein: impact on saccharomyces cerevisiae stress resistance. J. Photochem. Photobiol. B: Biol..

[bib0063] Ribeiro M.J., Reinders A., Boller T., Wiemken A., De Virgilio C. (1997). Trehalose synthesis is important for the acquisition of thermotolerance in Schizosaccharomyces pombe. Mol. Microbiol..

[bib0064] Ritchie M.E., Phipson B., Wu D., Hu Y., Law C.W., Shi W., Smyth G.K. (2015). limma powers differential expression analyses for RNA-sequencing and microarray studies. Nucleic. Acids. Res..

[bib0065] Robinson J.T., Thorvaldsdottir H., Turner D., Mesirov J.P. (2023). igv.js: an embeddable JavaScript implementation of the Integrative Genomics Viewer (IGV). Bioinformatics.

[bib0066] Sanchez Y., Taulien J., Borkovich K.A., Lindquist S. (1992). Hsp104 is required for tolerance to many forms of stress. EMBO J..

[bib0067] Scholes A.N., Stuecker T.N., Hood S.E., Locke C.J., Stacy C.L., Zhang Q., Lewis J.A. (2024). Natural variation in yeast reveals multiple paths for acquiring higher stress resistance. BMC. Biol..

[bib0068] Sherman B.T., Hao M., Qiu J., Jiao X., Baseler M.W., Lane H.C., Imamichi T., Chang W. (2022). DAVID: a web server for functional enrichment analysis and functional annotation of gene lists (2021 update). Nucleic. Acids. Res..

[bib0069] Shui W., Xiong Y., Xiao W., Qi X., Zhang Y., Lin Y., Guo Y., Zhang Z., Wang Q., Ma Y. (2015). Understanding the mechanism of thermotolerance distinct from heat shock response through proteomic analysis of industrial strains of Saccharomyces cerevisiae. Mol. Cell Proteomics..

[bib0070] Święciło A. (2016). Cross-stress resistance in Saccharomyces cerevisiae yeast—new insight into an old phenomenon. Cell Stress Chaperones.

[bib0071] Szklarczyk D., Kirsch R., Koutrouli M., Nastou K., Mehryary F., Hachilif R., Gable A.L., Fang T., Doncheva N.T., Pyysalo S., Bork P., Jensen L.J., von Mering C. (2023). The STRING database in 2023: protein-protein association networks and functional enrichment analyses for any sequenced genome of interest. Nucleic. Acids. Res..

[bib0072] Tang D., Chen M., Huang X., Zhang Guicheng, Zeng L., Zhang Guangsen, Wu S., Wang Y. (2023). SRplot: a free online platform for data visualization and graphing. PLoS. One.

[bib0073] Teng X., Cheng W.-C., Qi B., Yu T.-X., Ramachandran K., Boersma M.D., Hattier T., Lehmann P.V., Pineda F.J., Hardwick J.M. (2011). Gene-dependent cell death in yeast. Cell Death. Dis..

[bib0074] The Alliance of Genome Resources Consortium (2024). Updates to the alliance of genome resources central infrastructure. Genetics.

[bib0075] Thomas P.D., Ebert D., Muruganujan A., Mushayahama T., Albou L.-P., Mi H. (2022). PANTHER: making genome-scale phylogenetics accessible to all. Protein Sci..

[bib0076] Verghese J., Abrams J., Wang Y., Morano K.A. (2012). Biology of the heat shock response and protein chaperones: Budding yeast (Saccharomyces cerevisiae) as a model system. Microbiol. Mol. Biol. Rev..

[bib0077] Wallace E.W., Kear-Scott J.L., Pilipenko E.V., Schwartz M.H., Laskowski P.R., Rojek A.E., Katanski C.D., Riback J.A., Dion M.F., Franks A.M., Airoldi E.M., Pan T., Budnik B.A., Drummond D.A. (2015). Reversible, specific, active aggregates of endogenous proteins assemble upon heat stress. Cell.

[bib0078] Walton E.F., Pringle J.R. (1980). Effect of growth temperature upon heat sensitivity in Saccharomyces cerevisiae. Arch. Microbiol..

[bib0079] Zhang B., Geberekidan M., Yan Z., Yi X., Bao J. (2023). Very high thermotolerance of an adaptive evolved Saccharomyces cerevisiae in cellulosic ethanol fermentation. Fermentation.

