# Supplementary material for: Disentangling non-specific and condition-specific transcriptomic responses: A framework from heat stress in yeast
Source: Curr Res Microb Sci. 2026 Jul 20;11:100649. doi: 10.1016/j.crmicr.2026.100649 (PMC13450573; doi:10.1016/j.crmicr.2026.100649)
Supplement: Supplementary file 1 [file mmc1.pdf]

Fig. S1

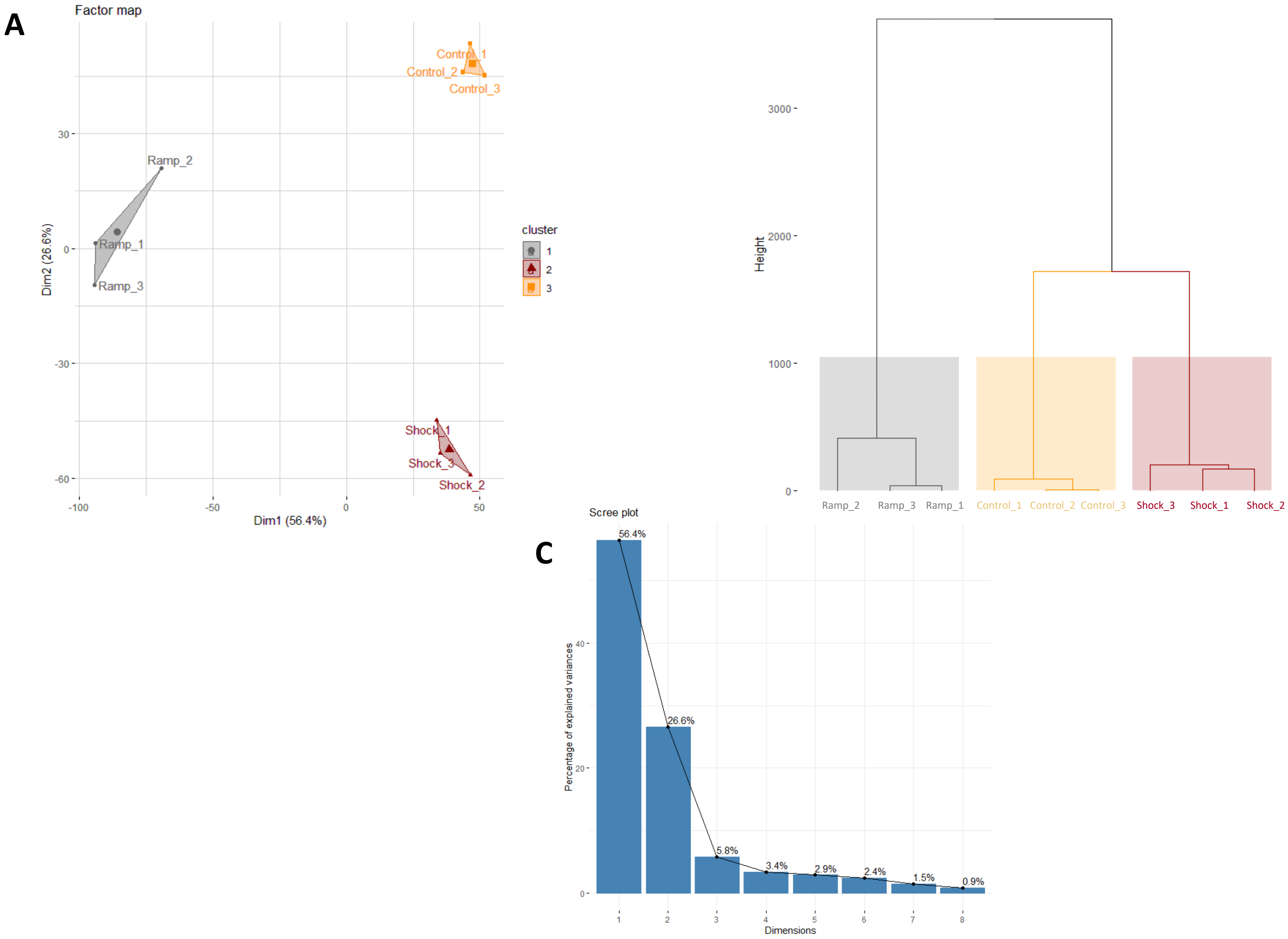
A
Factor map
30
0
-30
-60
-100
-50
0
50
Dim1 (56.4%)
Dim2 (26.6%)
Ramp_2
Ramp_1
Ramp_3
Control_1
Control_2
Control_3
Shock_1
Shock_3
Shock_2
cluster
1
2
3
B
Cluster Dendrogram
Height
3000
2000
1000
0
Ramp_2
Ramp_3
Ramp_1
Control_1
Control_2
Control_3
Shock_3
Shock_1
Shock_2
C
Scree plot
Percentage of explained variances
Dimensions
56.4%
26.6%
5.8%
3.4%
2.9%
2.4%
1.5%
0.9%
40
20
0
1
2
3
4
5
6
7
8
