# Supplementary material for: Disentangling non-specific and condition-specific transcriptomic responses: A framework from heat stress in yeast
Source: Curr Res Microb Sci. 2026 Jul 20;11:100649. doi: 10.1016/j.crmicr.2026.100649 (PMC13450573; doi:10.1016/j.crmicr.2026.100649)
Supplement: Supplementary file 2 [file mmc2.pdf]

Fig. S2

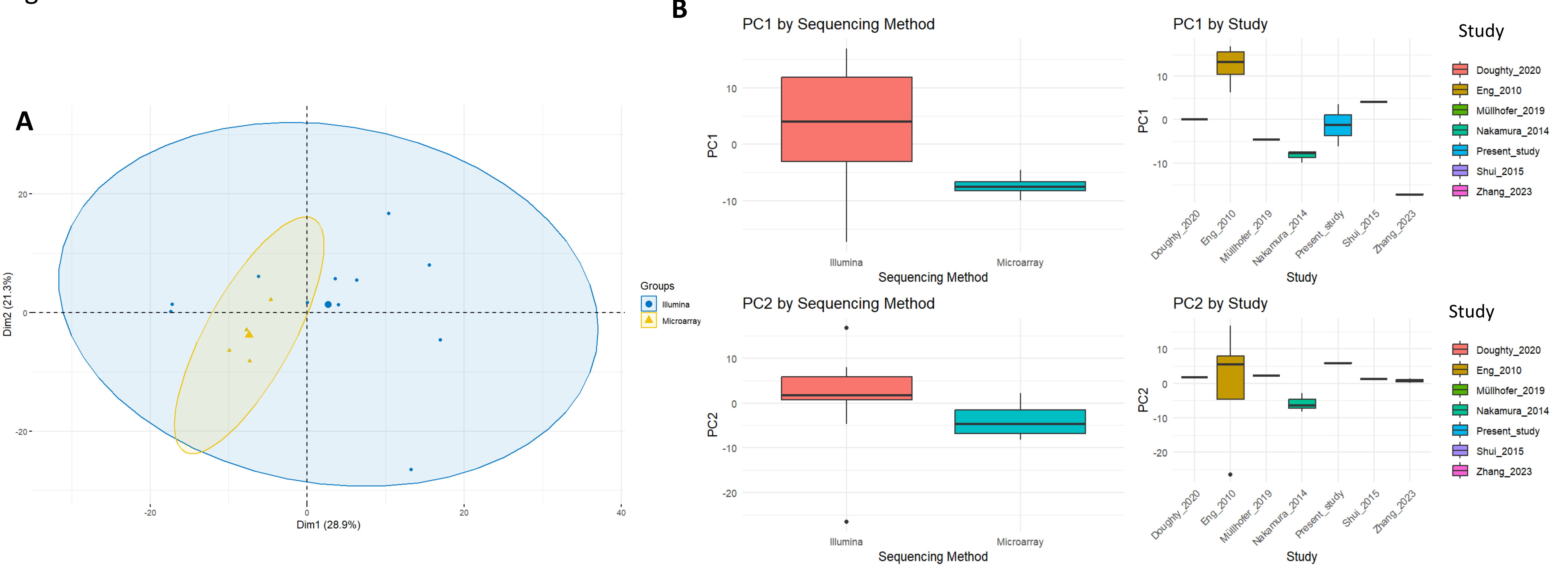


C

| Comparison | Shapiro-Wilk (residual normality) | Test used | Main test p-value | Variance test used | Variance test p-value |
|---|---|---|---|---|---|
| PC1 ~ Method | 0.0000067 (non-normal) | Kruskal-Wallis | 0.4334 | Fligner-Killeen | 0.2965 |
| PC1 ~ Study | 0.000036 (non-normal) | Kruskal-Wallis | 0.8987 | Fligner-Killeen | 0.297 |
| PC2 ~ Method | 0.044 (normal) | ANOVA | 0.421 | Levene’s test | 0.2501 |
| PC2 ~ Study | 0.15 (normal) | ANOVA | 0.926 | Levene’s test | 0.5788 |
