# Supplementary material for: Disentangling non-specific and condition-specific transcriptomic responses: A framework from heat stress in yeast
Source: Curr Res Microb Sci. 2026 Jul 20;11:100649. doi: 10.1016/j.crmicr.2026.100649 (PMC13450573; doi:10.1016/j.crmicr.2026.100649)
Supplement: Supplementary file 3 [file mmc3.pdf]

Fig. S3

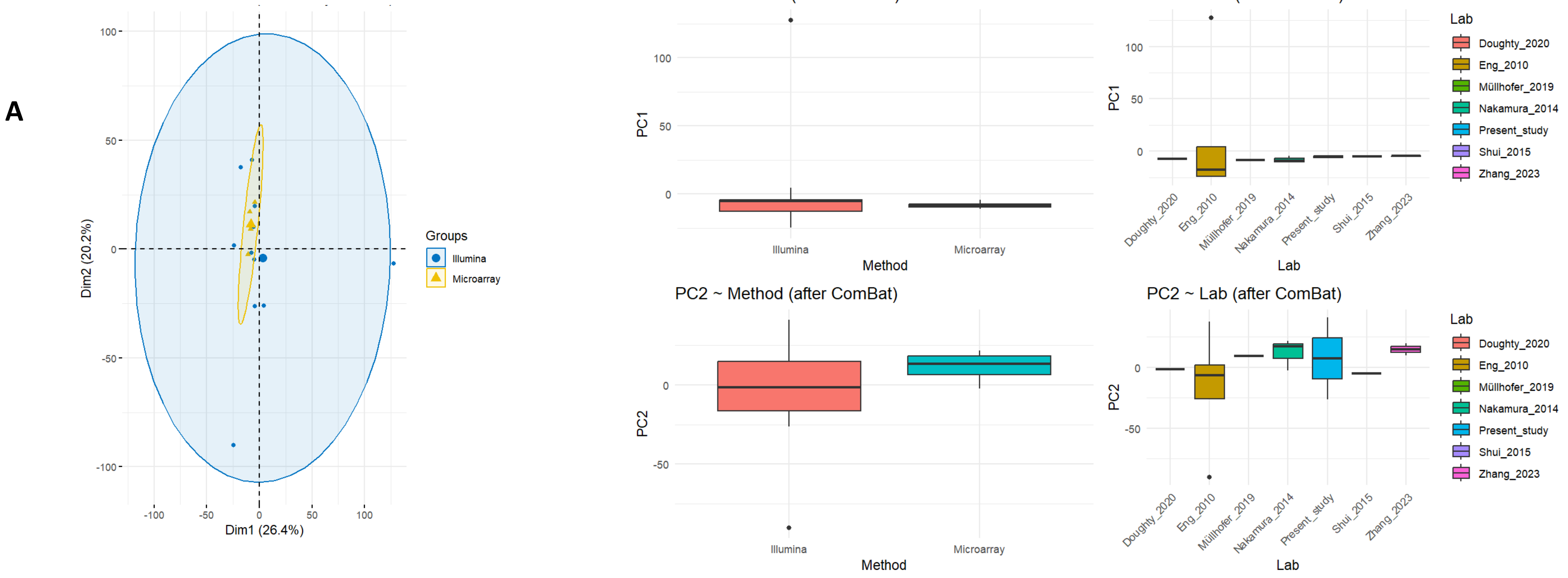

C

Comparison	Shapiro-Wilk (residual normality)	Test used	Main test p-value	Variance test used	Variance test p-value
PC1 ~ Method	0.0000067 (non-normal)	ruskal-Wall	0.4334	Fligner-Killeen	0.2965
PC1 ~ Lab	0.000036 (non-normal)	ruskal-Wall	0.8987	Fligner-Killeen	0.297
PC2 ~ Method	0.044 (normal)	ANOVA	0.421	Levene's test	0.2501
PC2 ~ Lab	0.15 (normal)	ANOVA	0.926	Levene's test	0.5788
