# Supplementary material for: Disentangling non-specific and condition-specific transcriptomic responses: A framework from heat stress in yeast
Source: Curr Res Microb Sci. 2026 Jul 20;11:100649. doi: 10.1016/j.crmicr.2026.100649 (PMC13450573; doi:10.1016/j.crmicr.2026.100649)
Supplement: Supplementary file 4 [file mmc4.pdf]

Fig. S4

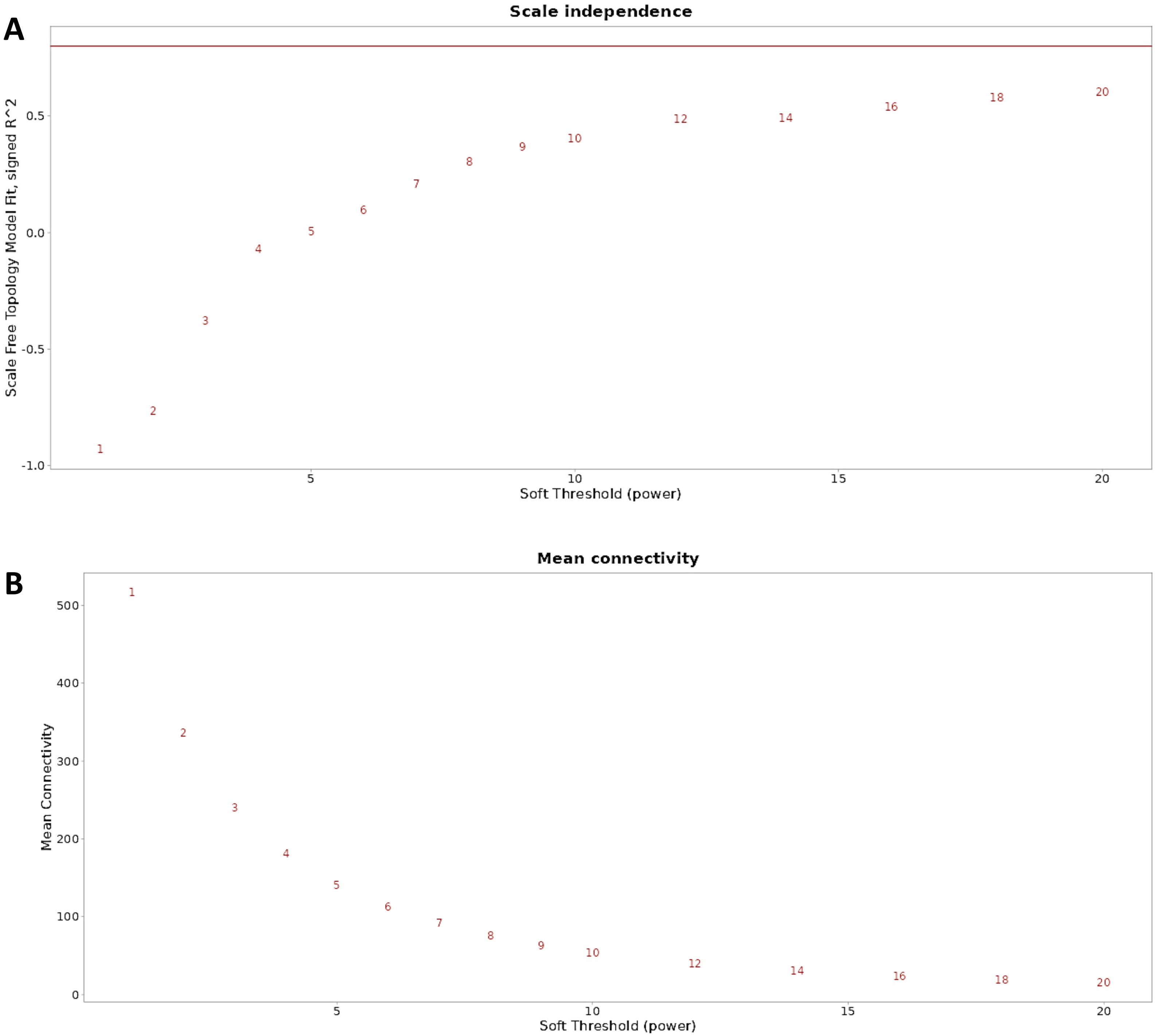
A
Scale independence
Scale Free Topology Model Fit, signed R^2
Soft Threshold (power)
B
Mean connectivity
Mean Connectivity
Soft Threshold (power)
