# Supplementary material for: Disentangling non-specific and condition-specific transcriptomic responses: A framework from heat stress in yeast
Source: Curr Res Microb Sci. 2026 Jul 20;11:100649. doi: 10.1016/j.crmicr.2026.100649 (PMC13450573; doi:10.1016/j.crmicr.2026.100649)
Supplement: Supplementary file 5 [file mmc5.pdf]

Fig. S5

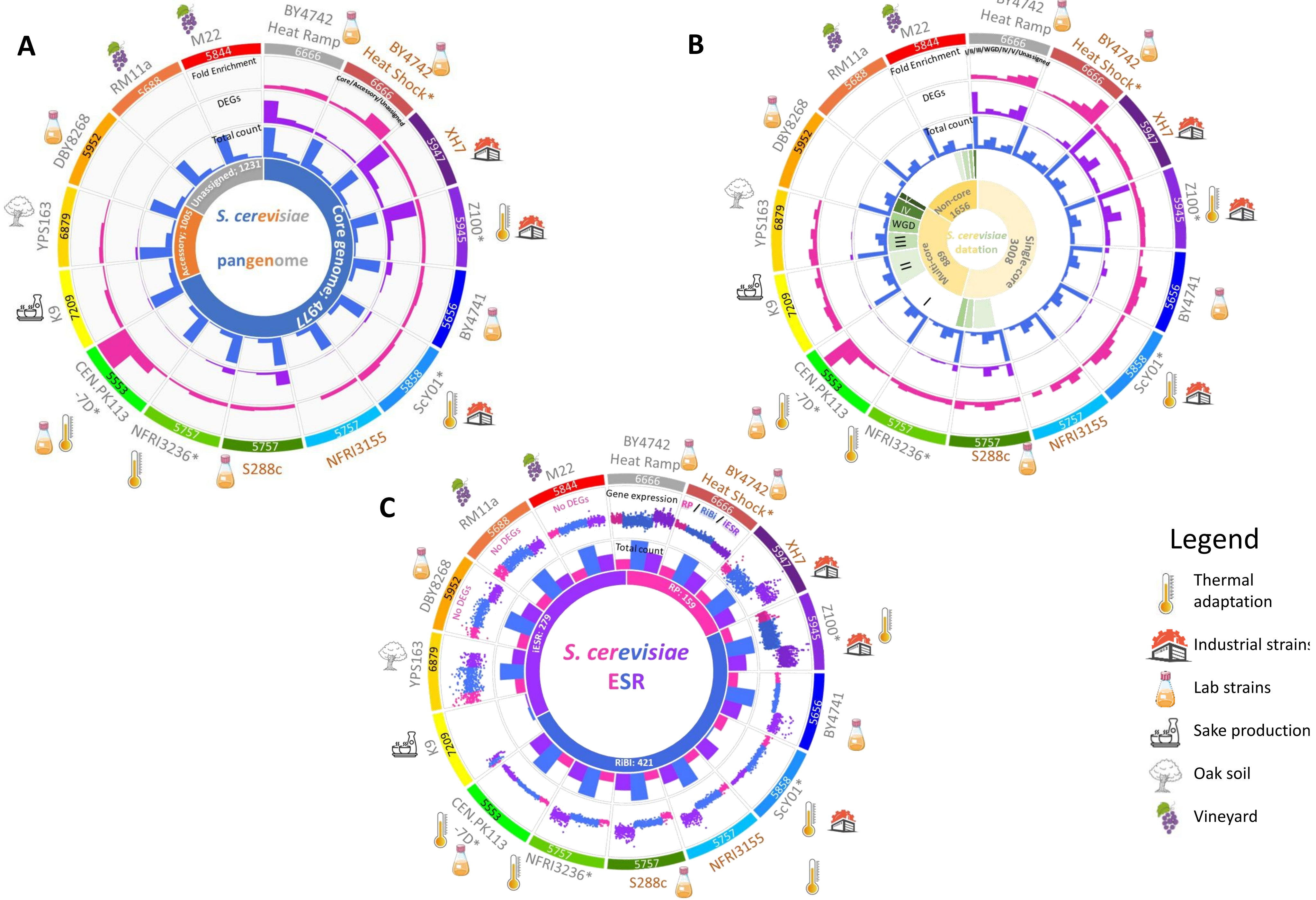

A
S. cerevisiae
pangenome
Core genome: 4977
Accessory: 1005
Unassigned: 1231
Fold Enrichment
DEGs
Total count
Core/Accessory/Unassigned
BY4742
Heat Ramp
6666
BY4742
Heat Shock*
XH7
5947
Z100*
5945
BY4741
5656
ScY01*
5858
NFRI3155
5757
S288c
5757
NFRI3236*
CEN.PK113
-7D*
5553
K9
7209
YPS163
6879
DBY8268
5952
RM11a
5688
M22
5844
B
S. cerevisiae
datation
Non-core
1656
Single-core
3008
Multi-core
889
WGD
IV
III
II
I
I/II/III/WGD/IV/V/Unassigned
C
S. cerevisiae
ESR
RP: 159
RiBi: 421
iESR: 279
Gene expression
RP / RiBi / iESR
No DEGs
Legend
Thermal
adaptation
Industrial strains
Lab strains
Sake production
Oak soil
Vineyard
