# Supplementary material for: Disentangling non-specific and condition-specific transcriptomic responses: A framework from heat stress in yeast
Source: Curr Res Microb Sci. 2026 Jul 20;11:100649. doi: 10.1016/j.crmicr.2026.100649 (PMC13450573; doi:10.1016/j.crmicr.2026.100649)
Supplement: Supplementary file 6 [file mmc6.pdf]

Fig. S6

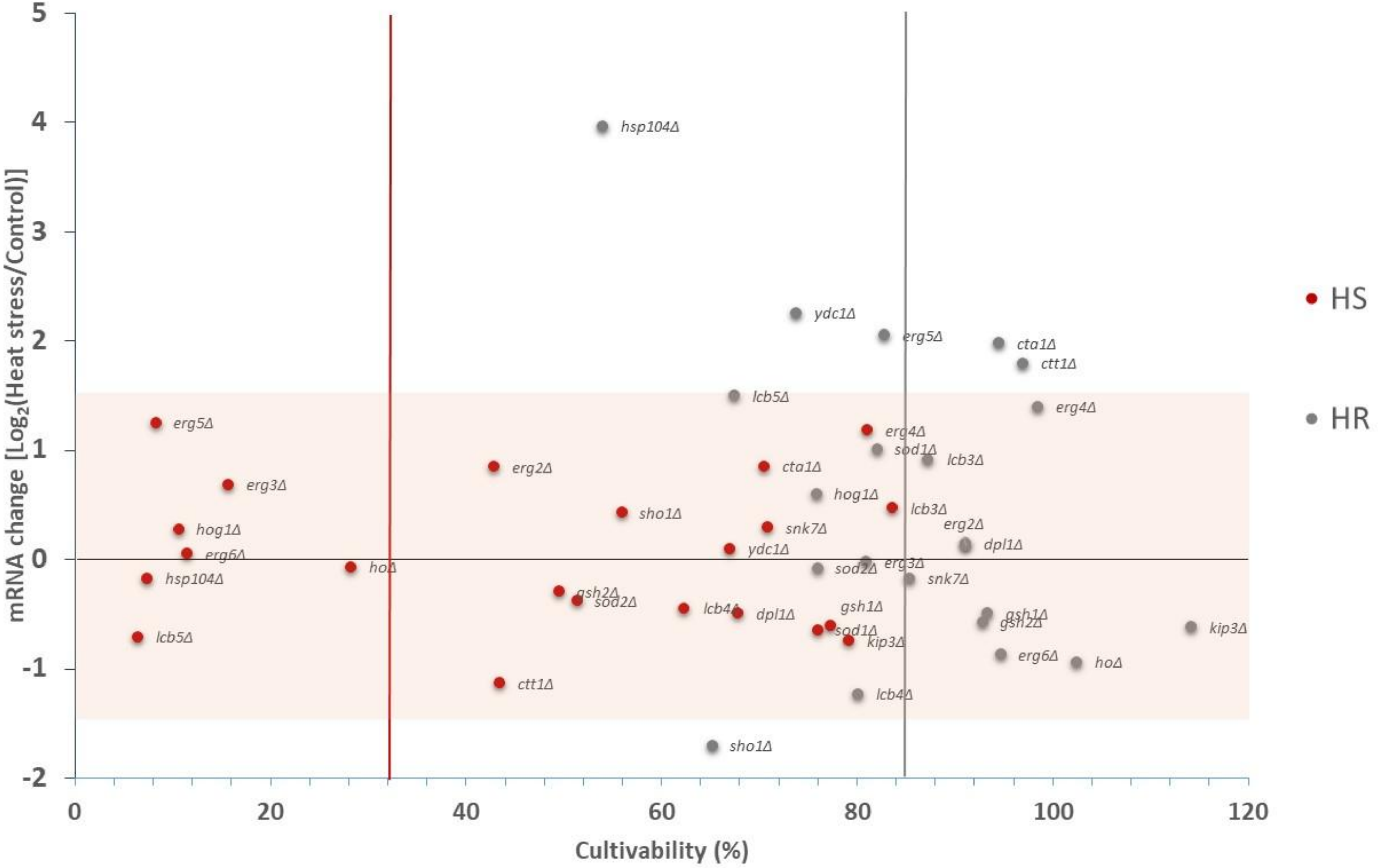

mRNA change [Log2(Heat stress/Control)]
Cultivability (%)
HS
HR
hsp104Δ
ydc1Δ
erg5Δ
cta1Δ
ctt1Δ
lcb5Δ
erg4Δ
erg5Δ
erg4Δ
sod1Δ
lcb3Δ
erg2Δ
cta1Δ
erg3Δ
hog1Δ
sho1Δ
lcb3Δ
hog1Δ
snk7Δ
erg2Δ
dpl1Δ
ydc1Δ
erg6Δ
hoΔ
sod2Δ
erg3Δ
snk7Δ
hsp104Δ
gsh2Δ
sod2Δ
lcb4Δ
dpl1Δ
gsh1Δ
gsh1Δ
gsh2Δ
kip3Δ
sod1Δ
lcb5Δ
kip3Δ
erg6Δ
hoΔ
ctt1Δ
lcb4Δ
sho1Δ
