# Supplementary material for: Disentangling non-specific and condition-specific transcriptomic responses: A framework from heat stress in yeast
Source: Curr Res Microb Sci. 2026 Jul 20;11:100649. doi: 10.1016/j.crmicr.2026.100649 (PMC13450573; doi:10.1016/j.crmicr.2026.100649)
Supplement: Supplementary file 7 [file mmc7.pdf]

Fig. S7

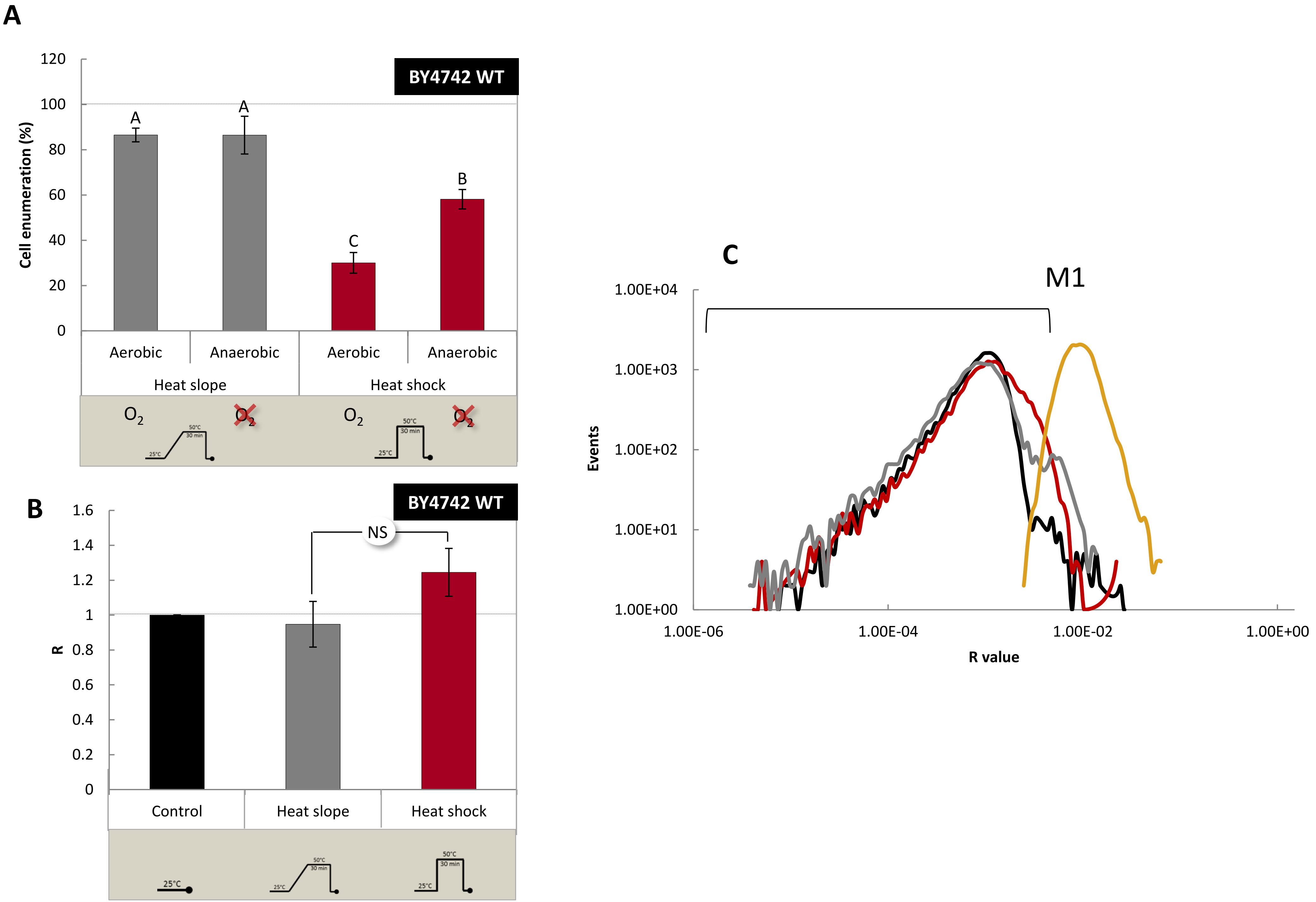
A
BY4742 WT
Cell enumeration (%)
120
100
80
60
40
20
0
A
A
C
B
Aerobic
Anaerobic
Aerobic
Anaerobic
Heat slope
Heat shock
B
BY4742 WT
NS
R
1.6
1.4
1.2
1
0.8
0.6
0.4
0.2
0
Control
Heat slope
Heat shock
25°C
C
M1
Events
1.00E+04
1.00E+03
1.00E+02
1.00E+01
1.00E+00
1.00E-06
1.00E-04
1.00E-02
1.00E+00
R value
