# Supplementary material for: Disentangling non-specific and condition-specific transcriptomic responses: A framework from heat stress in yeast
Source: Curr Res Microb Sci. 2026 Jul 20;11:100649. doi: 10.1016/j.crmicr.2026.100649 (PMC13450573; doi:10.1016/j.crmicr.2026.100649)
Supplement: Supplementary file 8 [file mmc8.docx]

**Supplementary Materials and Methods**

**Oxygraphy.** Immediately after heat stress treatments, five 100 µL samples from the same culture were pooled and placed in the chamber at 25°C of an oxygraph equipped with a Clark-type O_2_ electrode (Hansatech Instruments Ltd., Norfolk, UK) containing 500 µL of YPD liquid medium. By using the software, Oxygraph Plus V1.01 (Hansatech Instruments Ltd.), the respiration rates were recorded continuously for 15 min.

**Cell enumeration.** The colony forming unit method (CFU) was used to enumerate yeasts. Immediately after the application of the heat stresses (or after washing samples with fresh YPD medium in the case of control samples), 10 µL of four replicates of the appropriate decimal dilution of the cell suspension were dropped on YPD-agar medium. CFU were counted after incubation for 48h at 25 °C. At least four independent cultures of yeast were tested.

**Anaerobic treatments.** The steps of cell washing, heat treatments and dilution of the cell suspension were done in oxygen-free YPD obtained by bubbling nitrogen for four hours.

**Yeast KO mutant strains.** The haploid strain of *Saccharomyces cerevisiae* BY4742 (*MATα*; *his3Δ1*; *leu2Δ0*; *lys2Δ0*; *ura3Δ0*) was used as wild-type strain (**Table S17**). Its entire isogenic knockout mutants (**Table S17**) were purchased from Euroscarf (Frankfurt, Germany).

**Circos visualizations of the transcriptomic features of 15 strains of *S. cerevisiae*.** Circos visualizations (**Fig. S5**) were generated using the online software shinyCircos (<https://venyao.xyz/shinyCircos-V1/>) (Yu et al., 2018). Fold enrichment is calculated using **Equation 1** where dg represents the total number of differentially expressed genes in group or cluster g, ng represents the total number of genes in group or cluster g, D represents the total number of differentially expressed genes across all groups or clusters and N represents the total number of genes across all groups or clusters. This formula compares the proportion of DEGs within a specific group to the proportion of DEGs across all analysed gene groups. A fold enrichment greater than 1 indicates that DEGs are more concentrated in the group of interest than would be expected by chance, suggesting potential biological relevance. Conversely, a fold enrichment less than 1 indicates under-representation. This method of calculation was based on that previously described by Doughty et al. (2020).

$Fold enrichment=\frac{\left( \frac{d_{g}}{n_{g}} \right)}{\left( \frac{D}{N} \right)}$	(**Equation 1**)

**References**

Doughty, T.W., Domenzain, I., Millan-Oropeza, A., Montini, N., de Groot, P.A., Pereira, R., Nielsen, J., Henry, C., Daran, J.-M.G., Siewers, V., Morrissey, J.P., 2020. Stress-induced expression is enriched for evolutionarily young genes in diverse budding yeasts. Nat Commun 11, 2144. https://doi.org/10.1038/s41467-020-16073-3

Yu, Y., Ouyang, Y., Yao, W., 2018. shinyCircos: an R/Shiny application for interactive creation of Circos plot. Bioinformatics 34, 1229–1231. https://doi.org/10.1093/bioinformatics/btx763
